# Developing guidance for a risk-proportionate approach to blinding statisticians within clinical trials: a mixed methods study

**DOI:** 10.1186/s13063-022-06992-5

**Published:** 2023-01-31

**Authors:** Mais Iflaifel, Kirsty Sprange, Jennifer Bell, Andrew Cook, Carrol Gamble, Steven A. Julious, Edmund Juszczak, Louise Linsell, Alan Montgomery, Christopher Partlett

**Affiliations:** 1 Nottingham Clinical Trials Unit, University of Nottingham, Nottingham, UK; 2 National Perinatal Epidemiology Unit, Nuffield Department of Population Health, University of Oxford, Oxford, UK; 3 University of Southampton, Southampton, UK; 4 Liverpool Clinical Trials Centre, University of Liverpool, Liverpool, UK; 5 School of Health and Related Research (ScHARR), University of Sheffield, Sheffield, UK

**Keywords:** Blinding, Statisticians, Mixed methods, Stakeholder meeting, Clinical trials, Clinical trials unit

## Abstract

**Background:**

Existing guidelines recommend statisticians remain blinded to treatment allocation prior to the final analysis and that any interim analyses should be conducted by a separate team from the one undertaking the final analysis. However, there remains substantial variation in practice between UK Clinical Trials Units (CTUs) when it comes to blinding statisticians. Therefore, the aim of this study was to develop guidance to advise CTUs on a risk-proportionate approach to blinding statisticians within clinical trials.

**Methods:**

This study employed a mixed methods approach involving three stages: (I) a quantitative study using a cohort of 200 studies (from a major UK funder published between 2016 and 2020) to assess the impact of blinding statisticians on the proportion of trials reporting a statistically significant finding for the primary outcome(s); (II) a qualitative study using focus groups to determine the perspectives of key stakeholders on the practice of blinding trial statisticians; and (III) combining the results of stages I and II, along with a stakeholder meeting, to develop guidance for UK CTUs.

**Results:**

After screening abstracts, 179 trials were included for review. The results of the primary analysis showed no evidence that involvement of an unblinded trial statistician was associated with the likelihood of statistically significant findings being reported, odds ratio (OR) 1.02 (95% confidence interval (CI) 0.49 to 2.13). Six focus groups were conducted, with 37 participants. The triangulation between stages I and II resulted in developing 40 provisional statements. These were rated independently by the stakeholder group prior to the meeting. Ten statements reached agreement with no agreement on 30 statements. At the meeting, various factors were identified that could influence the decision of blinding the statistician, including timing, study design, types of intervention and practicalities. Guidance including 21 recommendations/considerations was developed alongside a Risk Assessment Tool to provide CTUs with a framework for assessing the risks associated with blinding/not blinding statisticians and for identifying appropriate mitigation strategies.

**Conclusions:**

This is the first study to develop a guidance document to enhance the understanding of blinding statisticians and to provide a framework for the decision-making process. The key finding was that the decision to blind statisticians should be based on the benefits and risks associated with a particular trial.

**Supplementary Information:**

The online version contains supplementary material available at 10.1186/s13063-022-06992-5.

## Background

There is limited empirical evidence to guide trial teams within UK Clinical Trials Units (CTUs) about the practice of blinding statisticians. Although there are examples of published guidance on blinding statisticians in clinical trials in the US, they are not internationally adopted [1, 2]. A survey of the UK Clinical Research Collaboration (UKCRC) CTUs conducted by the authors in 2020 identified that there was considerable variation in practice when it came to blinding statisticians. The survey contained 20 respondents, all from different UK CTUs. Half of the respondents indicated the CTU had a fixed approach to blinding statisticians rather than assessing the risk according to the trial circumstances.

CTUs can be broadly split into those that always blind the statisticians and involve a second statistician for unblinded/disaggregate analyses [1] and those that maintain the blind until it is necessary to unblind statisticians (e.g. for a Data Monitoring Committee (DMC) analysis report). While there may be perceived benefits to maintaining the blind of statisticians, given the potential logistical and resource cost as well as other shortcomings of always blinding the statistician, it seems incongruent to apply this approach in all cases [2]. This could be seen as analogous to always insisting on performing a placebo-controlled trial.

There are clearly benefits to developing an evidence-based risk-proportionate approach to blinding statisticians in clinical trials, in line with other areas of clinical trial conduct [3]. The lack of an evidence-base to inform such a risk-proportionate approach motivated the Blinding of Trial Statisticians (BOTS) research team to develop guidance for CTUs on blinding statisticians in trials. The overarching aim of BOTS was to develop practical guidance for blinding trial statisticians (TSs) in clinical trials. In this study, TSs are the statisticians responsible for the day-to-day statistical input into the trial, conduct data cleaning, querying and analysis (usually under the supervision of a Senior Trial Statistician). Throughout we refer to ‘the TS’; however, the role of the TS might be undertaken by more than one individual.

The objectives of BOTS were to:

1. Compare the outcomes of recently published randomised trials where the statistician was blinded prior to the final analysis versus those where the statistician was not
2. Further explore current practice in academic CTUs, and reasons for these practices
3. Understand stakeholder views on important risks and benefits to consider when deciding on blinding practice
4. Provide recommendations and a practical tool to enable CTUs to utilise a risk-based approach when considering blinding of the TS

Objectives 2 and 3 are the focus of another qualitative study [4].

## Methods

### Design

BOTS employed a mixed methods approach conducted in three stages.

- Stage I: Quantitative data was obtained from randomised trials funded by the UK National Institute for Health and Care Research (NIHR) Health Technology Assessment (HTA) and the MRC-NIHR Efficacy and Mechanism Evaluation (EME) programmes to assess the impact of statistician blinding on the proportion of trials reporting statistically significant findings for the primary outcome(s).
- Stage II: Qualitative data was collected by conducting focus groups with key stakeholders who work in the delivery and oversight of clinical trials, in order to explore their perspectives on when/how to blind TSs.
- Stage III: This consisted of two parts: [1] A first draft of the provisional guidance statements was developed by analysing and comparing the findings of stages I and II [2]; A stakeholder meeting was held to review and refine the provisional guidance statements.

**Fig. 1:**
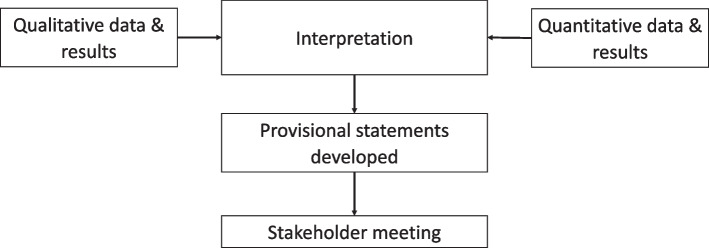
Developing the guidance document for blinding TSs in clinical trials

To develop a guidance document for blinding TSs in clinical trials, a triangulation design was used [5]. Triangulation enables comparison of concurrently collected data obtained via different methods to be explored for interaction, thereby adding validity to research findings [6]. This study follows the Good Reporting of A Mixed Methods Study (GRAMMS) guidelines [7]. Figure 1 illustrates the study methods used to develop the guidance document.

#### Stage I: Quantitative study

The NIHR Evaluation Trials and Studies Coordinating Centre (NETSCC) database was searched to identify publications of randomised controlled trials in the NIHR Journals Library between 1 January 2016 and 31 December 2020. Studies classified as pilot or feasibility were excluded. This cohort was chosen as it contained recently reported high-quality randomised trials with minimal potential for publication bias or other methodological deficiencies that could confound the comparison of interest.

##### Screening

The abstracts of potentially eligible studies published in this period (i.e. those that were flagged in the NETSCC database as being randomised controlled trials, funded by the HTA or EME Programme, and not marked as a pilot or feasibility study) were reviewed independently by two authors (CP & MI) to confirm eligibility.

##### Data collection

A data extraction form (see Additional file 1) was developed by CP and MI and reviewed by a third research team member (KS) and the study co-investigators (AM, EJ, LL, JB, CG, SJ and AK). The data extraction form was adopted from the Revised Cochrane risk-of-bias tool for randomised trials (RoB 2) [8] and used to extract data about trial characteristics associated with risk of bias. MI extracted data related to key study characteristics, e.g. trial design, number and type of interventions and comparators, number of study arms and primary outcomes. The extraction of these data was successfully piloted by a previous study [9]. CP extracted data related to the type of assessed outcomes (e.g. binary, continuous and time-to-event), whether any imputation was performed for the primary outcome(s), the treatment effect measure and effect size. CP and MI independently extracted data on the blinding status of TSs and whether a statistically significant finding was reported for the primary outcome. The latter was based on whether the study authors claimed statistically significant findings with respect to at least one primary outcome for at least one primary comparison (rather than based on the reported *p*-value).

For trials with a single primary comparison (e.g. two-arm trials with a single primary outcome), the reported *p*-value was also independently extracted. Independent data extraction was additionally performed for data that might influence the findings of a trial, such as the amount of missing primary outcome data, presence of allocation concealment and the blinding status of the participants, clinicians and outcome assessors. The extracted data were then compared, and discrepancies resolved via an agreement between the two researchers. Where a resolution could not be reached, a third researcher (KS) was invited to arbitrate.

Where the blinding status of the TS was not clear in a monograph, the research team contacted the named statistician(s) and/or manager to provide information about the blinding status of the TS in the clinical trial.

##### Analysis

Descriptive statistics describing the study characteristics were presented by blinding status of TSs. Continuous data were summarised in terms of the mean, standard deviation, median, lower and upper quartiles, minimum, maximum and number of observations. Categorical data were summarised in terms of frequency counts and percentages. No formal statistical comparisons were made.

The proportion of statistically significant findings for the primary outcome was compared between the included studies where TSs were blinded versus not blinded, using a logistic regression model, adjusting for whether there were multiple comparisons, whether the target sample size was achieved, and the blinding status of participants, clinicians and outcome assessors. These covariates were included based on the prior belief that these factors might plausibly influence the likelihood of statistically significant findings being reported and might also be associated with the blinding status of the statistician (thereby confounding the association of interest). A trial was determined to include multiple comparisons if the trial included either (a) more than two treatment groups or (b) multiple co-primary outcomes. In the primary analysis model, a trial was determined to be not blinded if any of the participant, clinician or outcome assessor were not blinded. To facilitate inclusion in the statistical model, where the blinding status of any of these groups was unclear, this was assumed as not blinded. A secondary analysis model included blinding status of each of participants, clinicians and outcome assessors, as separate covariates. In addition, the sensitivity of the findings to alternative model specification was also explored. In particular, the primary analysis was repeated using models that additionally adjusted for the proportion of missing data for the primary outcome, as well as models determined using forwards selection and backwards elimination of covariates.

The between-group effect was reported using an adjusted odds ratio along with a *p*-value and a corresponding 95% confidence interval. The primary analysis included only those studies where the blinding status of the TS could be confirmed. However, a sensitivity analysis included all studies, by assuming that the TS was not blinded for those studies where the blinding status was unclear.

For trials with a single primary comparison, the reported *p*-values were compared between groups by using a beta-regression model, adjusting for whether the target sample size was achieved and the blinding status of participants, clinicians and outcome assessors.

#### Stage II: Qualitative study

The aim of the qualitative stage of BOTS was to explore the experiences, opinions and ideas of key stakeholders on blinding statisticians in clinical trials. Therefore, it was relevant to conduct focus groups with key stakeholders including statisticians, Trial Steering Committee (TSC) and DMC members and those working within trial management who have experience in the delivery and oversight of clinical trials. Further details about the methods of this stage are the focus of a separate publication [4].

#### Stage III: Guidance development

##### Overview

**Fig. 2:**
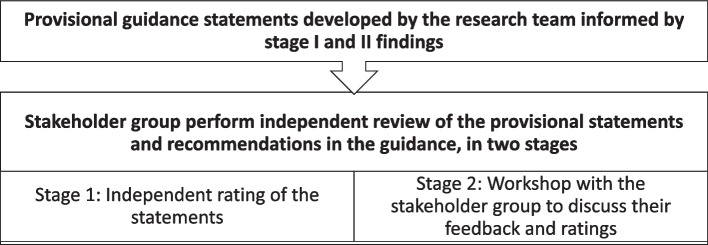
Summary of the stakeholder group meeting

The results from stages I and II were triangulated to develop provisional statements to advise CTUs on a risk-proportionate approach to blinding statisticians within trials. The provisional statements were developed by the research team using an iterative process in partnership with a stakeholder group, comprised of the BOTS study co-investigators and an invited multidisciplinary group of experts. Figure 2 provides a summary of the stakeholder group meeting. While the original intention was that the stakeholder group would take on the role of a consensus group, responsible for formally approving/agreeing the content of the guidance document, the findings of the qualitative study indicated that firm guidance would not be achievable and formal consensus building methodology would not be appropriate. Instead, the focus of the stakeholder meeting was to reflect upon on the statements and discuss and agree the important factors that might influence the decision to blind or not blind the statistician.

##### Participant identification

The research team sent an invitation letter to potential participants of the stakeholder group via email to the UKCRC, the Trials Methodology Research Partnership (TMRP) and UK Trial Managers’ Network (UKTMN), supplemented by personal invitations suggested by the study co-investigators.

##### Pre-meeting survey

Prior to the meeting, the research team circulated a survey to the stakeholder group members containing provisional guidance statements (see Additional file 2), along with a document explaining the purpose of the survey and instructions for completion. Stakeholders were requested to score their agreement with each provisional statement using a 9-point Likert scale ranging from [1] strongly disagree to [9] strongly agree.

Based on our experience with the qualitative sub-study [4], in which focus group participants strongly suggested that firm guidance was not likely to be achievable or helpful, the aim of the survey was to quantify the extent to which the stakeholder group agreed or disagreed with the proposed statements. As a result, we proposed broad classifications of agreement and disagreement to categorise the participants’ ratings, as summarised in Table 1.

**Table 1:** Classification﻿ of the provisional statements

Category	Classification rule
High agreement	>70% agree (score 7,8,9)
Low agreement	>50% agree (score 7,8,9)
No agreement	No majority for agree or disagree
Low disagreement	>50% disagree (score 1,2,3)
High disagreement	>70% disagree (score 1,2,3)

Participants were able to add comments/feedback on the provisional statements in the survey. Confidentiality of responses was ensured; individual responses were known only to the research team. Participants were asked to complete their ratings for the guidance statements a week before the meeting to enable the research team to collate and summarise the responses and to present and discuss them on the day.

##### Stakeholder meeting

The online stakeholder meeting comprised a morning and an afternoon session. Participants were allocated to one of three breakout rooms (five to six participants/room), facilitated by CP, AM and EJ. Each room started and finished on a different topic to ensure that at least one survey section was covered comprehensively by each group. Verbal consent was obtained from participants before starting to record the meeting.

The statements were organised under two themes originally identified from the qualitative study (Stage II). The ‘Processes’ theme included statements related to the timing of unblinding, impact on statistician interaction with other groups and practicalities. The ‘Trial design and conduct’ theme included statements focused on study design, type of analysis, interventions and outcomes.

Statements where there was high agreement were presented to the participants but not discussed during the meeting. All other statements were discussed to determine whether the statements should be refined and added to the guidance or omitted completely. The stakeholder group were also invited to discuss and agree what factors (if any) would change the participants’ initial score. Feedback on the main discussion points raised during the meeting were tabulated and iterative meetings between the research team (CP, KS and MI) were conducted to discuss the participants’ feedback in order to rephrase, refine or remove statements. The revised statements were circulated to the BOTS study co-investigators for them to comment and agree on the final statements’ structure and clarity.

## Results

### Stage I: Quantitative study

**Fig. 3:**
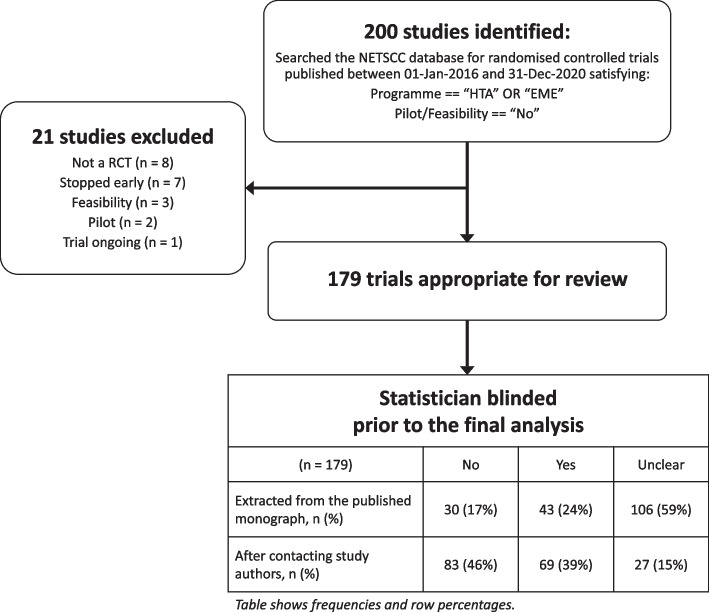
Study flow diagram

After screening the abstracts of the 200 studies, 21 studies were excluded leaving a total of 179 trials appropriate for inclusion in the review. The study flow diagram is shown in Fig. 3. A list of the included/excluded studies is included as part of Additional file 3. Following data extraction, the blinding status of the statistician remained unclear for 106 (59%) of the included studies. After contacting study authors where the blinding status was unclear, the blinding status of the statistician was determined in 152 (85%) of included studies.

The characteristics of the trials included in the review are described in Table 2 by blinding status of the statistician, while Table 3 describes the approach to allocation concealment and blinding of participants, clinicians and outcome assessors. Both Table 2 and Table 3 present row percentages as these report characteristics and design features of the trial that might possibly have influenced the blinding status of the statistician. For instance, data reported in Table 2 suggest that 33% of CTIMPs utilised a blinded statistician, compared with 42% of non-CTIMPs. Data reported in Table 3 suggest that the blinding status of statistician was not strongly associated with the blinding status of other groups.

**Table 2:** Characteristics of included trials by blinding status of the statistician

Characteristic	Statistician blinded prior to the final analysis			
	No (***N*** = 83)	Yes (***N*** = 69)	Unknown (***N*** = 27)	Total (***N*** = 179)
**Publication year,** ***n*** **(%)**				
2016	20 (56%)	12 (33%)	4 (11%)	36
2017	12 (43%)	11 (39%)	5 (18%)	28
2018	22 (58%)	13 (34%)	3 (8%)	38
2019	17 (44%)	15 (38%)	7 (18%)	39
2020	12 (32%)	18 (47%)	8 (21%)	38
**Journal,** ***n*** **(%)**				
HTA	64 (44%)	59 (41%)	22 (15%)	145
EME	19 (56%)	10 (29%)	5 (15%)	34
**Trial design,** ***n*** **(%)**				
Individually randomised	72 (46%)	60 (38%)	24 (15%)	156
Cluster	8 (50%)	7 (44%)	1 (6%)	16
Factorial	1 (25%)	2 (50%)	1 (25%)	4
Other	2 (67%)	0 (0%)	1 (33%)	3
**Interventions,** ***n*** **(%)**				
Drug	36 (49%)	25 (34%)	12 (16%)	73
Device	4 (44%)	2 (22%)	3 (33%)	9
Surgery	11 (69%)	1 (6%)	4 (25%)	16
Complex	34 (38%)	45 (51%)	10 (11%)	89
**CTIMP,** ***n*** **(%)**				
No	48 (44%)	46 (42%)	15 (14%)	109
Yes	35 (50%)	23 (33%)	12 (17%)	70
**Hypothesis testing framework,** ***n*** **(%)**				
Superiority	75 (46%)	66 (40%)	22 (13%)	163
Non-inferiority	8 (57%)	2 (14%)	4 (29%)	14
Equivalence	0 (0%)	1 (50%)	1 (50%)	2
**Treatment groups,** ***n*** **(%)**				
2	69 (48%)	54 (37%)	22 (15%)	145
>2	14 (41%)	15 (44%)	5 (15%)	34
**Placebo controlled,** ***n*** **(%)**				
No	60 (45%)	52 (39%)	20 (15%)	132
Yes	23 (49%)	17 (36%)	7 (15%)	47
**Multiple primary outcomes,** ***n*** **(%)**				
No	64 (44%)	60 (41%)	21 (14%)	145
Yes	19 (56%)	9 (26%)	6 (18%)	34
**Clinical area,** ***n*** **(%)**				
Blood	2 (67%)	0 (0%)	1 (33%)	3
Cancer	7 (64%)	2 (18%)	2 (18%)	11
Cardiovascular	5 (42%)	5 (42%)	2 (17%)	12
Generic health relevance	5 (36%)	6 (43%)	3 (21%)	14
Infection	12 (71%)	2 (12%)	3 (18%)	17
Inflammatory and immune system	2 (50%)	2 (50%)	0 (0%)	4
Injuries and accidents	5 (63%)	2 (25%)	1 (13%)	8
Mental health	6 (25%)	13 (54%)	5 (21%)	24
Metabolic and endocrine	5 (71%)	1 (14%)	1 (14%)	7
Musculoskeletal	2 (67%)	1 (33%)	0 (0%)	3
Neurological	6 (40%)	5 (33%)	4 (27%)	15
Oral and gastrointestinal	3 (43%)	2 (29%)	2 (29%)	7
Renal and urogenital	3 (50%)	2 (33%)	1 (17%)	6
Reproductive health and childbirth	8 (50%)	7 (44%)	1 (6%)	16
Respiratory	5 (50%)	5 (50%)	0 (0%)	10
Skin	2 (29%)	5 (71%)	0 (0%)	7
Stroke	4 (33%)	7 (58%)	1 (8%)	12
Other	1 (33%)	2 (67%)	0 (0%)	3

Table displays frequencies and row percentages

*CTIMP* Clinical Trials of Investigational Medicinal Products

**Table 3:** Allocation concealment and blinding by blinding status of the statistician

Characteristic	Statistician blinded prior to the final analysis			
	No (***N*** = 83)	Yes (***N*** = 69)	Unknown (***N*** = 27)	Total (***N*** = 179)
**Allocation concealed,** ***n*** **(%)**				
Yes	82 (46%)	69 (39%)	26 (15%)	177
*Unclear*	*1 (50%)*	*0 (0%)*	*1 (50%)*	*2*
**Participants blinded,** ***n*** **(%)**				
No	52 (44%)	45 (38%)	21 (18%)	118
Yes	30 (51%)	23 (39%)	6 (10%)	59
*Unclear*	*1 (50%)*	*1 (50%)*	*0*	*2*
**Clinician blinded,** ***n*** **(%)**				
No	56 (46%)	45 (37%)	22 (18%)	123
Yes	26 (49%)	22 (42%)	5 (9%)	53
*Unclear*	*1 (33%)*	*2 (67%)*	*0*	*3*
**Outcome assessor blinded,** ***n*** **(%)**				
No	23 (51%)	16 (36%)	6 (13%)	45
Yes	50 (43%)	48 (41%)	19 (16%)	117
*Unclear*	*10 (59%)*	*5 (29%)*	*2 (12%)*	*17*

Table displays frequencies and row percentages

Table 4 reports the outcomes of the included trials, including the reporting of statistically significant findings for the primary outcome. Table 4 presents column percentages, as these are outcomes of the trial that might possibly be influenced by the blinding status of the statistician. There does not appear to be any association between the reporting of statistically significant findings and the blinding status of the statistician; however, trials that utilised a blinded statistician appeared to recruit to target more frequently, have more missing data and utilise imputation methods more frequently.

**Table 4:** Outcomes of the included trials

	Statistician blinded prior to the final analysis		
	No (***N***=83)	Yes (***N***=69)	Unclear (***N***=27)
**Statistically significant finding reported for primary outcome,** ***n/N*** **(%)**	23/83 (28%)	19/69 (28%)	8/27 (30%)
**Sample size planned**			
Mean [SD]	1686.1 [5326.6]	1178.6 [2110.7]	1151.7 [1313.9]
Median [25th, 75th centile]	540 [240, 900]	480 [312, 1200]	544 [308, 1180]
**Sample size achieved**			
Mean [SD]	1647 [5433.7]	1304.7 [2888.8]	1116 [1424.4]
Median [25th, 75th centile]	540 [269, 1010]	475 [300, 1023]	487 [247, 1211]
**Target sample size achieved,** ***n/N*** **(%)**	56/83 (67%)	52/69 (75%)	15/27 (56%)
**Percentage of target sample size achieved**			
Mean [SD]	98.2 [25.7]	100.7 [24.8]	99 [60.1]
Median [25th, 75th centile]	100.6 [97.4, 105.8]	101 [100, 107.4]	100 [83.2, 102.6]
**Percentage of primary outcome data missing**			
Mean [SD]	9.3 [11.4]	13.7 [10.4]	14.8 [15.6]
Median [25th, 75th centile]	4 [1, 16]	11.5 [6, 20]	9 [3, 23]
*Unclear, n*	*1*	*3*	*0*
**Missing data imputed for primary outcome,** ***n/N*** **(%)**	16/82 (20%)	20/65 (31%)	10/26 (38%)
*Unclear, n*	*1*	*4*	*1*

Unless otherwise specified the table displays frequencies and column percentages

*SD* standard deviation

The results of the primary analysis, investigating the influence of the blinding status of the statistician on the reporting of statistically significant findings, are presented in Table 5. Including only those trials where the blinding status of the statistician could be confirmed (*n*=152), there was no evidence that involvement of an unblinded trial statistician was associated with the likelihood of significant findings being reported, odds ratio (OR) 1.02 (95% confidence interval (CI) 0.49 to 2.13). However, there was strong evidence that failure to blind any of clinicians, participants and outcome assessors increased the likelihood of statistically significant findings being reported, OR 3.00 (95% CI 1.16 to 7.78). The findings remain consistent when imputing missing data for the blinding status of the statistician.

**Table 5:** Primary analysis—odds ratios (OR) describing the influence of the blinding status of the statistician and other selected study design features on whether a statistically significant finding is reported

**Blinding status of the statistician known**		
*N* = 152	**OR (95% CI)**^a^	***p*-value**
Statistician not blinded	1.02 (0.49, 2.13)	0.958
Multiple comparisons^b^	1.34 (0.63, 2.86)	0.450
Target sample size achieved	1.15 (0.51, 2.62)	0.736
Trial not blinded^c^	3.00 (1.16, 7.78)	0.024
**Imputation of missing blinding status of the statistician**^d^		
*N* = 179	**OR (95% CI)**^a^	***p*-value**
Statistician not blinded	1.04 (0.52, 2.08)	0.907
Multiple comparisons^b^	1.38 (0.69, 2.78)	0.360
Target sample size achieved	1.40 (0.66, 2.95)	0.383
Trial not blinded^c^	2.76 (1.14, 6.69)	0.025

^a^Odds ratio (and 95% confidence interval) from logistic regression model comparing the influence of the listed study design features on the likelihood of a statistically significant finding

^b^A trial is defined as having multiple comparisons if it contains more than two treatment groups or multiple co-primary outcomes

^c^A trial is defined as not blinded if any of the participants, clinicians and outcome assessors are not blinded. To facilitate inclusion in the model wherever the blinding status is unclear, this was assumed as not blinded

^d^Where blinding status is unclear it was assumed the statistician was not blinded

The sensitivity of the findings to alternative model specification was also explored and conclusions were found to be consistent. Table A1 in the Additional file 4 reports the model output for an alternative model where blinding status of participants, clinicians and outcome assessors were included as separate covariates. The results were also found to be consistent for alternative model specification. The findings were unchanged for models that additionally adjusted for the proportion of missing data for the primary outcome, as well as models determined using forwards selection and backwards elimination of covariates (further details also included in Additional file 4).

Table A2 in the Additional file 4 reports the findings of the secondary analysis describing the influence of the blinding status of the statistician and other selected study design features on those studies where it was possible to obtain a single reported *p*-value (*n* = 110). There was little evidence to suggest that the blinding status of the statistician was associated with the reported *p*-value.

### Stage II: Qualitative study

Six focus groups were conducted with 37 participants taking part (average of 6 per group). FGs included a mixture of statisticians, trial managers, data managers and data coordinators. Four themes were identified from the analysis of the FGs’ transcripts:

1. Statistical models of work: six models were identified where all models shared the involvement of at least two statisticians, one typically more junior TS and one more senior.
2. Factors affecting the decision to blind or not blind statisticians: a range of factors were highlighted such as types of data, study design, outcomes, analysis, interventions and some external factors, e.g. funder limitations and expectations and personal relationships with sponsors and chief investigators.
3. Benefits of blinding versus not blinding TSs: The benefits of blinding the TS suggested by the FGs participants included reducing the perception of bias, and reducing pressure on statistician to reveal data. The main benefits of not blinding were understanding data in context which leads to higher quality analysis and reduces the risk of incorrect decision-making and erroneous assumptions about the data.
4. Practicalities: How to maintain the blind of the statistician during trial delivery, issues of staffing and unit capacity and the level of statisticians’ experience and knowledge about blinding were challenging factors raised by participants across all the FGs that might influence the practicality of blinding the TS.

More detailed results of this stage can be found in a separate publication [4].

### Stage III: Stakeholder meeting

#### Triangulation and pre-meeting activities

The triangulation between stage I and II findings resulted in the highlighting of two main themes: the influence of statisticians’ blinding status on trial findings, and the association of statisticians’ blinding status with trial characteristics, e.g. study design, type of intervention. Interestingly, there was convergence between the quantitative and qualitative findings when it came to the impact of blinding of statisticians on findings. The statistician’s blinding status had no significant impact on trial outcomes. Almost all participants in the FGs’ study agreed that they did not feel statisticians knowingly introduced bias to the clinical trial [4]. Another convergence between the two data sets concerned the type of comparison; having a blinded statistician was more common in superiority trials than in non-inferiority studies and FG participants indicated that blinding the statistician in superiority trials was regarded as more important than in non-inferiority trials. Participants in FGs felt that having a blinded TS was more important for Clinical Trials of Investigational Medicinal Products (CTIMPs). This result is somewhat at odds with the quantitative data, which found that the proportion of blinded statisticians was higher (42%) in non-CTIMPs compared to CTIMPs (33%). However, the apparent lower rate of blinding of TSs in CTIMPs observed in the quantitative study is driven predominantly by unclear reporting of TS blinding in open-label CTIMPs (7/24).

The triangulation method led to a better understanding of the research question and resulted in the development of 40 draft provisional statements, which were sent to the stakeholder meeting participants for them to independently rate prior to the meeting day. All stakeholder attendees were invited to rate the provisional statements. The overall response to the survey was good with 80% of the participants independently rating the provisional statements. Table 6 shows the number of statements in each category. A summary of the responses for each statement is provided in Additional file 5.

**Table 6:** Number of provisional statements in each category

Category	Number of statements
High agreement	10
Low agreement	7
No agreement	21
Low disagreement	2
High disagreement	0

The majority of the statements did not reach agreement in either direction; however, there was unanimous agreement that the decision to blind or not blind the statistician should be based on the benefits and risks associated with a particular trial. It was clear from the free text feedback that agreement or disagreement with the proposed statements on blinding TSs was dependent on contextual factors, i.e. trial-specific and CTU-specific factors. Although the importance of contextual factors was the prominent issue raised by the participants in the free text feedback, analysing participants’ feedback identified other important themes to be discussed in the stakeholder meeting. For example, practical mitigation strategies against the risks of blinding or not blinding the statistician, blinding statisticians in adaptive designs, credibility of pseudo-blinding/partial blinding (i.e. using coded groups) and issues related to the marketing authorisation and whether it should be used as a consideration to blind or not blind statisticians.

#### Stakeholder meeting

The stakeholder meeting was held in November 2021. A virtual stakeholder meeting via Microsoft Teams was held with 16 stakeholders from the following roles: statisticians, methodologists, trial management and data management professionals, CTU directors and unit managers, as well as members of independent trial oversight committees and representatives from the NIHR and the MHRA. A list of stakeholders’ names, organisations and roles is detailed in Additional file 6.

The responses to the provisional statements from the online survey provided a rich source of material for further discussion with the participants in the stakeholder meeting. During the meeting, there was a general feeling between the participants that the provisional statements in the survey were broad and not specific. Almost all participants agreed that it was crucial not to use definitive statements but to favour softer ones to accommodate flexibility. The stakeholder meeting was in many ways successful, with agreement emerging about the various factors that could influence the decision whether to blind the statistician. These factors, also identified during the qualitative phase, were categorised under seven broad themes: ‘timing of unblinding’, ‘ impact on statistician interaction with other groups’, ‘study design’, ‘types of intervention’, ‘types of outcome’, ‘additional roles and responsibilities’ and ‘practicalities’.

Participants agreed that timing of unblinding is a key factor; for example, unblinding the TS after the statistical analysis plan (SAP) has been approved mitigates against some of the risk of the TS introducing any bias, while maintaining the blind until after the final database lock and approval of the SAP almost completely eliminates the risk. It was also identified that the blinding status of TSs could impact on their interactions with other members of the trial team and oversight committees. Regarding study design, there was also agreement that the risk of bias associated with unblinding TSs was likely to be smaller for open-label trials, where the nature of the treatments under investigation may not permit blinding and other members of the research team are unblinded [10]. Finally, the resources required to maintain the blind of TSs need to be proportionate to the perceived benefit, to justify blinding the statistician.

#### Post-meeting: finalising recommendations and considerations

**Fig. 4:**
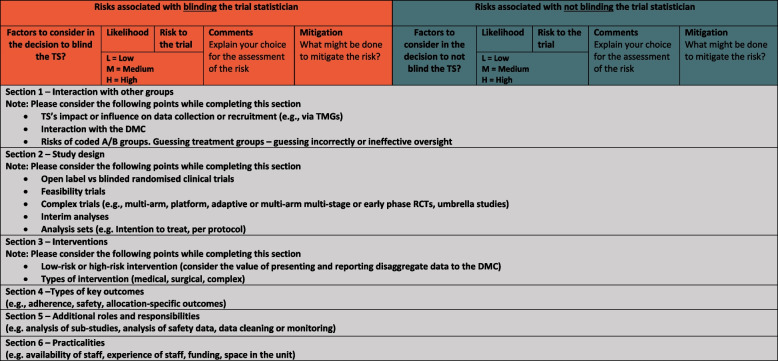
Headers and sections of the BOTS Risk Assessment Tool

The revision of the provisional statements, in line with the stakeholders meeting output, resulted in developing a guidance document for achieving a risk-proportionate approach to blinding statisticians within clinical trials. The guidance document consisted of 21 final recommendations/considerations categorised under seven sections. Further justification and explanation are included for each recommendation/consideration in the guidance document (Additional file 7). The overall recommendation that the guidance highlighted was ‘The decision to blind or not blind the statistician should be based on the benefits and risks associated with a particular trial’. Based on the participants’ recommendation, the research team developed the BOTS Risk Assessment Tool (BRAT). The BRAT provides CTUs with a framework for assessing risks associated with blinding/not blinding the statisticians and for developing appropriate mitigation strategies. The tool is split into 6 sections (see Fig. 4), and each section includes rows with suggestions for potential risks. For more information about proposed questions for potential risks in each section, see Additional file 8.

The guidance document also includes details about models for DMC interaction; benefits, risks and mitigation strategies related to the decision to blind or not blind statisticians; and the BRAT. Both the guidance document and the BRAT are freely available via the Nottingham Clinical Trials Unit BOTS study webpage [11].

## Discussion

### Key findings

Three key findings emerged from the BOTS study:

1. No evidence was found to show that the blinding status of the TS was associated with the likelihood of significant findings being reported for the primary outcome.
2. A trial’s design and characteristics can influence the decision about whether to blind TSs.
3. There is strong support within UK CTUs for a risk-proportionate approach to blinding statisticians within clinical trials. The BOTS study found that a broad multidisciplinary group of stakeholders were unanimous in agreeing that the decision to blind or not blind the TS should be based on the risks associated with a given trial.

#### No evidence that blinding status of the trial statistician is associated with reporting statistically significant findings

The finding that the blinding status of TSs was not associated with the reporting of statistically significant findings does not support the approach in existing guidelines [12, 13], which recommend to maintain the blind of TSs to treatment allocation until the final analysis, and to conduct any interim analysis by a separate team of statisticians or programmers to those who will be involved in the final analysis. However, consistent with the literature [14, 15], this study found a strong correlation between the clinicians’, participants’ and outcome assessors’ blinding status and the significance of the findings reported. It is worth noting that reporting of blinding methodology is often absent or of low quality in published articles of RCTs [16, 17], a shortcoming confirmed by the present study. Only 41% of the included studies documented the blinding status of TSs, the majority unsurprisingly relating to cases in which the statistician was blinded.

#### Trial design and characteristics influence the decision to blind TSs

Most participants in the qualitative study [4] felt that it is more important to blind the TS in CTIMPs than in non-CTIMPs. This might be explained by the fact that current guidelines do not make a clear distinction between blinded statisticians and other personnel included in the trial (participants or clinicians). Consequently, participants in FGs for example assumed that CTIMPs needed more consideration with respect to blinding TSs, because of the frequent monitoring and auditing processes conducted by the MHRA. In practice, our findings showed that the statisticians were blinded more often in non-CTIMPs. This, in turn, might be explained by more frequent safety monitoring either causing or necessitating unblinding of the TS.

The finding that blinding of TSs is more prevalent (and considered more important) in superiority trials is contrary to previous studies that have suggested that the methods of blinding in equivalence or non-inferiority trials should mirror as much as possible those used in superiority trials, in order to remove factors that may obscure any differences between the study arms [18, 19].

#### Taking a risk-proportionate approach to blinding

There was strong agreement within the stakeholder group on the need for a risk-proportionate approach to deciding whether to blind TSs. Specifically, it was agreed that the decision should weigh up the benefits and risks associated with a particular trial. The importance of this concept is substantiated by other empirical evidence of the MHRA initiative for CTIMP trials, which highlighted that not all trials are equal and some clinical trials pose only a minimal additional risk in relation to safety and data integrity [20]. This initiative found that using a risk-proportionate approach could optimise the utilisation of resources and reduce the burden on the trials team, while still maintaining the quality and accuracy of trial results.

Little evidence was found in the literature search on the question of when and how to blind statisticians in clinical trials. Researchers have previously advocated blinding statisticians by using the independent statistician model [1] or by keeping statisticians reporting to data monitoring committees independent of the trial leadership and sponsor [2]. However, these recommendations are not based on empirical evidence and have not been universally adopted. Moreover, the DAMOCLES study group found that, while the independent statistician model has merit in some cases, the potential for loss of insight means that this approach is not recommended in general [21].

#### Development of guidance and a risk assessment tool

In response to these findings, guidance was developed based on the experiences of stakeholders and the findings from the quantitative and qualitative data, encompassing recommendations and considerations on when and how to blind TSs in clinical trials.

The recommendations and considerations, along with the BRAT, are designed to be flexible and can be implemented at different stages throughout the life cycle of a trial. For example, one could implement at either (a) the grant application stage to ensure appropriate funding is requested for the trial, or (b) during the trial set-up phase to determine the appropriate allocation of statistical resources. The BRAT could also be applied mid-study when determining the advantages and disadvantages of unblinding the TS (for instance, for an unplanned interim analysis). Both the guidance document and BRAT are designed to be used by the TS, preferably with input from the wider trial team.

While it is possible that the BRAT may not eliminate variation in practice, nor is it designed to, it will hopefully serve to ensure that the practice is justified and carefully considered. Crucially, it should also reduce the implementation of unnecessary procedures that cost money and time and can have unintended consequences.

### Strengths and limitations

The trials included in the quantitative study were limited to a cohort of the HTA and EME-funded trials, published between 2016 and 2020; however, this cohort provided high-quality trials that should be less prone to methodological deficiencies that could confound the comparison of interest.

A persistent challenge was achieving consistency in definition of statistician blinding. For instance, although guidance was issued by the BOTS study team, heterogeneity in the definition of statistician blinding applied by individual trial teams in the quantitative included studies was inevitable. Similarly, although the research team provided FG participants with a clear definition of statistician blinding, achieving consistency between participants within and across FGs remained a challenge.

The use of a mixed methods approach provided a comprehensive insight into the impact of the blinding status of TSs as the data was collected and triangulated from two sources. The qualitative data is likely representative of practice in UK CTUs as it was collated from a multidisciplinary and geographically diverse sample of key stakeholders, who had extensive experience in the delivery and oversight of clinical trials. Consequently, the findings are likely to be readily generalisable to practice within UK CTUs; however, it is unclear the extent to which these findings are applicable to other settings. For instance, within pharmaceutical-led trials (especially where the aim is to apply for market authorisation) or in low-resource settings, a risk-proportionate approach to blinding may not be appropriate. This is because the decision to blind or not may be pre-determined by other circumstances (e.g. regulatory or resource constraints). Nonetheless, in such settings, a careful exposition of the risks (and associated mitigation strategies) of blinding or not blinding remains valuable.

## Conclusion

The overarching purpose of the BOTS study was to develop guidance for CTUs to utilise a risk-based approach when deciding whether to blind statisticians in clinical trials. The blinding status of the statistician appeared to depend on certain trial characteristics, e.g. study design, type of comparison and the blinding status of other groups within the trial. No evidence was found to support the assertion that the blinding status of the statistician influenced reported findings. However, the risk of bias certainly appeared lower compared with unblinding participants, clinicians or outcome assessors, who perhaps have a greater opportunity to influence the findings. The guidance should prove useful in expanding the understanding of how and when to blind TSs and to help trial teams to use the BRAT in the decision-making process. We believe that using the guidance along with BRAT lays the groundwork for clinical trialists to apply evidence-based decision-making regarding blinding of statisticians.

## Supplementary Information

**Additional file 1.** Data extraction form.

**Additional file 2. **Online survey.

**Additional file 3.** A list of the included/excluded studies in the quantitative study.

**Additional file 4.** Additional analyses.

**Additional file 5.** BOTS stakeholder survey responses.

**Additional file 6.** BOTS stakeholder group membership.

**Additional file 7. **BOTS guidance.

**Additional file 8.** BOTS Risk Assessment Tool (BRAT).

## Data Availability

Data collected as part of the quantitative study are available from the corresponding author upon reasonable request. Requests will be subject to approval by a data sharing committee at the Nottingham Clinical Trials Unit. Data collected as part of the qualitative study are not available to be shared to preserve the anonymity of participants.

## Abbreviations

BOTS: Blinding of Trial Statisticians
BRAT: BOTS Risk Assessment Tool
CI: Chief Investigator
CRF: Case Report Form
CTIMPs: Clinical Trials of Investigational Medicinal Products
CTU: Clinical trials unit
DMC: Data Monitoring Committee
DMEC: Data Monitoring and Ethics Committee
DSMC: Data and Safety Monitoring Committee
EME: Efficacy and Mechanism Evaluation
FDA: Food and Drug Administration
FG: Focus group
HTA: Health Technology Assessment
MHRA: Medicines and Healthcare products Regulatory Agency
MRC: Medical Research Council
NIHR: Institute for Health and Care Research
NETSCC: NIHR Evaluation Trials and Studies Coordinating Centre
PI: Principal Investigator
RCT: Randomised clinical trial
RoB 2: Version 2 of the Cochrane tool for assessing risk of bias in randomised trials
SAP: Statistical analysis plan
TMG: Trial Management Group
TMRP: Trials Methodology Research Partnership
TS: Trial statistician
TSC: Trial Steering Committee
UKCRC: UK Clinical Research Collaboration
UKTMN: UK Trial Managers’ Network
