# Supplementary material for: Developing guidance for a risk-proportionate approach to blinding statisticians within clinical trials: a mixed methods study
Source: Trials. 2023 Jan 31;24:71. doi: 10.1186/s13063-022-06992-5 (PMC9887916; doi:10.1186/s13063-022-06992-5)
Supplement: Supplementary file 1 — Additional file 1. Data extraction form. [file 13063_2022_6992_MOESM1_ESM.docx]

**Additional file 1: Data Extraction Form**

BOTS

Quantitative Part

**Part 1: Trial’s Characteristics**

| **Variable** | **Variable type (responses)** | **Comments** | **Extracted by** |
| --- | --- | --- | --- |
| Trial NIHR number | Numeric | Already part of the extracted data | n/a |
| Journal | Free text | Already part of the extracted data | n/a |
| Publication date | Free text | Already part of the extracted data | n/a |
| Trial title | Free text | Already part of the extracted data | n/a |
| Trial design | Drop down (Individually randomised parallel-group trial, Cluster-randomised parallel-group trial, Factorial, Others)  If others, Free text | Monograph | MI |
| Trial’s clinical area | Free text (based on the UKCRC Health Research Classification System) | Monograph | MI |
| Type of interventions | **Drug:** Drop down (Yes, No)  **Surgery:** Drop down (Yes, No)  **Device:** Drop down (Yes, No)  **Complex interventions (behaviour, diet, others):**  Drop down (Yes, No) |  | MI |
| If the type of intervention was drug or device, was the trial CTIMP? | Drop down (Yes, No) | Check for evidence if the trial is under MHRA and registered on Eudract by checking the report, study protocol and the Eudract website | MI |
| Type of comparison | Drop down (Superiority, Non-inferiority, Equivalence) | Monograph | MI |
| Number of study arms | Numeric | Monograph | MI |
| Number of comparisons | Drop down (Single, Multiple) |  | MI |
| Was the comparator a placebo? | Drop down (Yes, No) |  | MI |
| Number of primary outcomes | Numeric |  | MI |
| Type of the assessed outcome | **Binary** Drop down (Yes, No)  **Continuous** Drop down (Yes, No)  **Time to event** Drop down (Yes, No)  **Others** |  | CP |
| Was there a statistical difference in the primary outcome? | Drop down (Yes, No) |  | MI & CP |
| P-value | Numeric | Only extracted for single comparison | MI & CP |
| Effect size | Numeric | Only extracted for single comparison | CP |
| Was the analysing statistician blinded prior to the final analysis? | Drop down (Yes, No, Unclear) | Monograph, if not available, we could contact the authors or the CTU | MI & CP |

MI – Mais Iflaifel, CP – Christopher Partlett

**Part 2: Domains that might influence the final outcome of a trial**

| **Domain** | **Questions** | **Response** | **Clarification Comments** | **Extracted by** |
| --- | --- | --- | --- | --- |
| **Randomisation process** | Was the allocation sequence random?  Was the allocation sequence concealed until participants were enrolled and assigned to interventions? | Drop down (Yes, No, Unclear)  Drop down (Yes, No, Unclear) |  | MI & CP |
| **Deviations from intended number of participants** | Number of planned participants?  Number of actual randomised participants? | Numeric  Numeric |  | MI |
| **Missing outcome data** | What % of primary outcome was missed?  Were any data imputed for the primary outcome? | Numeric  Drop down (Yes, No, Unclear) |  | MI & CP  CP |
| **Measuring outcome** | Were the participants blinded?  Were the treating clinicians blinded?  Were the people who collected the primary outcome data blinded? | Drop down (Yes, No, Unclear)  Drop down (Yes, No, Unclear)  Drop down (Yes, No, Unclear) |  | MI & CP |

MI – Mais Iflaifel, CP – Christopher Partlett
