# Supplementary material for: Developing guidance for a risk-proportionate approach to blinding statisticians within clinical trials: a mixed methods study
Source: Trials. 2023 Jan 31;24:71. doi: 10.1186/s13063-022-06992-5 (PMC9887916; doi:10.1186/s13063-022-06992-5)
Supplement: Supplementary file 2 — Additional file 2. Online survey. [file 13063_2022_6992_MOESM2_ESM.pdf]

Guidance for blinding statisticians in clinical trials

* If you are unable to answer a question, please leave the linear scale blank.
* If you have no preference in either direction, please choose 5 which indicates neither agree nor disagree.

*Required

1. Name *

Section 1: Timing

2. 1.1) The trial statistician should not produce the randomisation list/codes.

Mark only one oval.

	1	2	3	4	5	6	7	8	9	
Strongly disagree	◯	◯	◯	◯	◯	◯	◯	◯	◯	Strongly agree

3. 1.2) The statistician(s) drafting or reviewing the statistical analysis plan should remain blinded prior to the statistical analysis plan being approved.

Mark only one oval.

	1	2	3	4	5	6	7	8	9	
Strongly disagree	◯	◯	◯	◯	◯	◯	◯	◯	◯	Strongly agree

4. 1.3) The trial statistician can request access to treatment allocation codes after the statistical analysis plan being approved, prior to database lock.

Mark only one oval.

	1	2	3	4	5	6	7	8	9	
Strongly disagree	◯	◯	◯	◯	◯	◯	◯	◯	◯	Strongly agree

5. 1.4) The trial statistician should not have access to treatment allocation codes until after the database has been locked.

Mark only one oval.

	1	2	3	4	5	6	7	8	9	
Strongly disagree	◯	◯	◯	◯	◯	◯	◯	◯	◯	Strongly agree

6. 1.5) The trial statistician should remain blinded until after they have completed programming the final analysis

Mark only one oval.

	1	2	3	4	5	6	7	8	9	
Strongly disagree	◯	◯	◯	◯	◯	◯	◯	◯	◯	Strongly agree

7. 1.6) If possible, maintain the blind of all statisticians until the end of the study (for instance, if there are no interim analyses requiring disaggregate data).

Mark only one oval.

	1	2	3	4	5	6	7	8	9	
Strongly disagree	◯	◯	◯	◯	◯	◯	◯	◯	◯	Strongly agree

8. If you don't agree with any statement in Section 1, please explain why or suggest modifications

Section 2: Interaction with other groups

9. 2.1) It is important that the trial statistician attends the closed session of the Data Monitoring Committee (DMC).

Mark only one oval.

	1	2	3	4	5	6	7	8	9	
Strongly disagree	◯	◯	◯	◯	◯	◯	◯	◯	◯	Strongly agree

10. 2.2) It is beneficial to have at least two statisticians to prepare a Data Monitoring Committee (DMC) report (1) One non-blinded who conducts analysis by treatment group (2) One blinded involved in day-to-day management.

Mark only one oval.

	1	2	3	4	5	6	7	8	9	
Strongly disagree	◯	◯	◯	◯	◯	◯	◯	◯	◯	Strongly agree

11. 2.3) A statistician should remain blinded if they could impact or influence data collection or recruitment (e.g., via participation in or contribution to the discussions within the Trial Management Group (TMG)).

Mark only one oval.

	1	2	3	4	5	6	7	8	9	
Strongly disagree	◯	◯	◯	◯	◯	◯	◯	◯	◯	Strongly agree

12. 2.4) The Data Monitoring Committee (DMC) requires disaggregate data (by treatment group) to fulfil its role adequately.

Mark only one oval.

	1	2	3	4	5	6	7	8	9	
Strongly disagree	◯	◯	◯	◯	◯	◯	◯	◯	◯	Strongly agree

13. 2.5) Coded groups are more of a hindrance than a benefit for allowing the Data Monitoring Committee (DMC) to make effective decisions, (e.g., it can encourage the practice of guessing treatment groups, which risks inefficient or ineffective oversight).

Mark only one oval.

	1	2	3	4	5	6	7	8	9	
Strongly disagree	◯	◯	◯	◯	◯	◯	◯	◯	◯	Strongly agree

14. 2.6) If the focus of interim reports to the Data Monitoring Committee (DMC) are to describe data completeness or provide descriptive analyses (as opposed to comparative analyses) it is less important for the trial statistician to be blinded

Mark only one oval.

	1	2	3	4	5	6	7	8	9	
Strongly disagree	◯	◯	◯	◯	◯	◯	◯	◯	◯	Strongly agree

15. 2.7) For certain situations, blinded statisticians pose more of a risk to the trial (e.g., through suboptimal oversight and data monitoring) than having a non-blinded trial statistician.

Mark only one oval.

	1	2	3	4	5	6	7	8	9	
Strongly disagree	◯	◯	◯	◯	◯	◯	◯	◯	◯	Strongly agree

16. 2.8) For the trial statistician to conduct effective data cleaning and monitoring tasks, they need to be unblinded.

Mark only one oval.

	1	2	3	4	5	6	7	8	9	
Strongly disagree	◯	◯	◯	◯	◯	◯	◯	◯	◯	Strongly agree

17. If you don't agree with any statement in Section 2, please explain why or suggest modifications

Section 3: Study design

18. 3.1) It is not necessary to blind statisticians for feasibility studies.

Mark only one oval.

	1	2	3	4	5	6	7	8	9	
Strongly disagree										Strongly agree

19. 3.2) If there is a planned interim analysis, then it is preferable to have a blind and non-blinded statistician.

Mark only one oval.

	1	2	3	4	5	6	7	8	9	
Strongly disagree										Strongly agree

20. 3.3) If a trial is open-label, it is not necessary to blind the trial statistician.

Mark only one oval.

	1	2	3	4	5	6	7	8	9	
Strongly disagree										Strongly agree

21. 3.4) Pseudo-blinding the trial statistician (via the use of coded group names) is not feasible when there is an unequal allocation ratio.

Mark only one oval.

	1	2	3	4	5	6	7	8	9	
Strongly disagree	◯	◯	◯	◯	◯	◯	◯	◯	◯	Strongly agree

22. 3.5) For an adaptive trial where treatment groups may be added or dropped throughout the study (e.g. multi-arm multi-stage design) the trial statistician should not be blinded.

Mark only one oval.

	1	2	3	4	5	6	7	8	9	
Strongly disagree	◯	◯	◯	◯	◯	◯	◯	◯	◯	Strongly agree

23. 3.6) If the purpose of a trial is to apply for regulatory approval, it is important for the trial statistician to be blinded to treatment allocation.

Mark only one oval.

	1	2	3	4	5	6	7	8	9	
Strongly disagree	◯	◯	◯	◯	◯	◯	◯	◯	◯	Strongly agree

24. If you don't agree with any statement in Section 3, please explain why or suggest modifications

Section 4: Types of analysis

25. 4.1) It is less important to blind statistician where the primary analysis is intention-to-treat (ITT) as opposed to, for example, a per protocol analysis – where a subset of randomised participants is analysed.

Mark only one oval.

	1	2	3	4	5	6	7	8	9	
Strongly disagree										Strongly agree

26. 4.2) Trial statisticians should be unblinded for the purposes of monitoring treatment adherence.

Mark only one oval.

	1	2	3	4	5	6	7	8	9	
Strongly disagree										Strongly agree

27. 4.3) It is impractical to blind trial statisticians if they are required to analyse mechanistic sub-studies or investigate the action of the treatment prior to the final analysis.

Mark only one oval.

	1	2	3	4	5	6	7	8	9	
Strongly disagree										Strongly agree

28. If you don't agree with any statement in Section 4, please explain why or suggest modifications

Section 5: Types of intervention

29. 5.1) For interventions with a distinctive side effect profile, it is impractical to blind the statistician.

Mark only one oval.

	1	2	3	4	5	6	7	8	9	
Strongly disagree										Strongly agree

30. 5.2) For low-risk interventions (where disaggregate data are not require for monitoring safety) unblinding the statistician before the end of the trial is not necessary.

Mark only one oval.

	1	2	3	4	5	6	7	8	9	
Strongly disagree										Strongly agree

31. If you don't agree with any statement in Section 5, please explain why or suggest modifications

Section 6: Type of outcomes

32. 6.1) The statistician is unlikely to be able to influence the reporting of simple-to-derive ‘hard’/objective outcomes.

Mark only one oval.

	1	2	3	4	5	6	7	8	9	
Strongly disagree										Strongly agree

33. 6.2) If the outcome involves combining data from multiple sources, there is potentially a greater risk associated with having a non-blinded trial statistician

Mark only one oval.

	1	2	3	4	5	6	7	8	9	
Strongly disagree										Strongly agree

34. 6.3) Where ongoing analysis of safety outcomes is important, the trial statistician should not be blinded (either they will not have access to important safety data or will become unblinded because of safety data).

Mark only one oval.

	1	2	3	4	5	6	7	8	9	
Strongly disagree										Strongly agree

35. If you don't agree with any statement in Section 6, please explain why or suggest modifications

Section 7: Practicalities

36. 7.1a) If you need to blind a trial statistician, then you should maintain the blind by having rigorous processes for a blinded statistician to access/request data or treatment allocation.

Mark only one oval.

	1	2	3	4	5	6	7	8	9	
Strongly disagree										Strongly agree

37. 7.1b) If you need to blind a trial statistician, then you should maintain the blind by ensuring that access to certain types of data is controlled/limited to non-blinded statisticians or other trial team members.

Mark only one oval.

	1	2	3	4	5	6	7	8	9	
Strongly disagree	◯	◯	◯	◯	◯	◯	◯	◯	◯	Strongly agree

38. 7.1c) If you need to blind a trial statistician, then you should maintain the blind by giving the responsibility to data management (or another team) to prepare reports and strip out data which could unblind.

Mark only one oval.

	1	2	3	4	5	6	7	8	9	
Strongly disagree	◯	◯	◯	◯	◯	◯	◯	◯	◯	Strongly agree

39. 7.1d) If you need to blind a trial statistician, then you should maintain the blind by ensuring the randomisation list/system is generated and maintained by another statistician or a separate team.

Mark only one oval.

	1	2	3	4	5	6	7	8	9	
Strongly disagree	◯	◯	◯	◯	◯	◯	◯	◯	◯	Strongly agree

40. 7.2) If you are unable to maintain the blind then it's a waste of resources. (e.g. more complex database builds, increased workloads for other teams, inefficiency created by incomplete datasets for trial statistician).

Mark only one oval.

	1	2	3	4	5	6	7	8	9	
Strongly disagree	◯	◯	◯	◯	◯	◯	◯	◯	◯	Strongly agree

41. 7.3) Where a non-blinded statistician is involved, they should attend the Data Monitoring Committee (DMC) meetings and must have sufficient experience and knowledge of the trial and methods.

Mark only one oval.

	1	2	3	4	5	6	7	8	9	
Strongly disagree										Strongly agree

42. 7.4) The trial statistician should remain blinded if data are required to be presented separately by treatment arm at any stage prior to the final analysis

Mark only one oval.

	1	2	3	4	5	6	7	8	9	
Strongly disagree										Strongly agree

43. 7.5) The Data Monitoring Committee (DMC) and Trial Steering Committee (TSC) should be consulted as early as possible to ask if they intend to see disaggregate data at any point in the trial.

Mark only one oval.

	1	2	3	4	5	6	7	8	9	
Strongly disagree										Strongly agree

44. 7.6) Where an unplanned disaggregate analysis is requested by the Data Monitoring Committee (DMC), the risk of unblinding the trial statistician should be assessed at the time the request is made.

Mark only one oval.

	1	2	3	4	5	6	7	8	9	
Strongly disagree										Strongly agree

45. If you don't agree with any statement in Section 7, please explain why or suggest modifications

Section 8: Best Practice

46. 8.1) The trial statistician should always be blinded to treatment allocation.

Mark only one oval.

	1	2	3	4	5	6	7	8	9	
Strongly disagree										Strongly agree

47. 8.2) The trial statistician should never be blinded to treatment allocation.

Mark only one oval.

	1	2	3	4	5	6	7	8	9	
Strongly disagree										Strongly agree

48. 8.3) The decision to blind or not blind the statistician should be based on the benefits and risks associated with a particular trial.

Mark only one oval.

	1	2	3	4	5	6	7	8	9	
Strongly disagree										Strongly agree

49. If you don't agree with any statement in Section 8, please explain why or suggest modifications

50. Do you have any ideas for additional statements to be added that are not currently included in the proposed guidance statements?

This content is neither created nor endorsed by Google.

Google Forms
