# Supplementary material for: Developing guidance for a risk-proportionate approach to blinding statisticians within clinical trials: a mixed methods study
Source: Trials. 2023 Jan 31;24:71. doi: 10.1186/s13063-022-06992-5 (PMC9887916; doi:10.1186/s13063-022-06992-5)
Supplement: Supplementary file 3 — Additional file 3. A list of the included/excluded studies in the quantitative study. [file 13063_2022_6992_MOESM3_ESM.docx]

**Additional file 3: A list of the included/excluded studies in the quantitative study**

|  | Trial NIHR number | Title | Journal | Include | Reason if not included |
| --- | --- | --- | --- | --- | --- |
|  | 13/26/01 | Multi-centre randomised controlled trial with economic evaluation and nested qualitative study comparing early structured physiotherapy versus manipulation under anaesthesia versus arthroscopic capsular release for patients referred to secondary care with a frozen shoulder (Adhesive Capsulitis) | HTA | Yes |  |
|  | 11/71/03 | Multicentre randomised trial of the effectiveness and cost-effectiveness of basic versus biofeedback-mediated intensive pelvic floor muscle training for female stress or mixed urinary incontinence (OPAL-Optimal PFMT for Adherence Long-term) | HTA | Yes |  |
|  | 14/140/61 | Examining the benefit of graduated compression stockings as an adjunct to low dose low molecular weight heparin in the prevention of venous thromboembolism in elective surgical inpatients identified as moderate or high risk for venous thromboembolism – a multi-centre randomised controlled trial | HTA | Yes |  |
|  | 13/155/05 | Helping people cope with temptations to smoke to reduce relapse: A factorial randomised controlled trial | HTA | Yes |  |
|  | 12/164/16 | Phase III trial in IntrahepaTic CHolestasis of pregnancy (ICP) to Evaluate urSodeoxycholic acid (UDCA) in improving perinatal outcomes | EME | Yes |  |
|  | 13/25/20 | A multi-centred RCT of an augmented exercise referral scheme using web-based behavioural support in individuals with metabolic, musculo-skeletal and mental health conditions | HTA | Yes |  |
|  | 12/196/08 | COmmunity based Rehabilitation after Knee Arthroplasty (CORKA) | HTA | Yes |  |
|  | 12/24/02 | Home Interventions and Light therapy for the treatment of vitiligo (HI-Light Vitiligo Trial) | HTA | Yes |  |
|  | 10/57/23 | Clarifying the management of men with recurrent urethral stricture disease: A randomised trial of open urethroplasty versus endoscopic urethrotomy (OPEN) | HTA | Yes |  |
|  | 06/35/99 | INTERVAL Dental Recalls Trial [Investigation of NICE Technologies for Enabling Risk-Variable-Adjusted-Length Dental Recalls Trial] - Full Trial Follow-on | HTA | Yes |  |
|  | 13/52/04 | A multi-centre randomised controlled trial of the efficacy and mechanism of action of gabapentin for the management of chronic pelvic pain in women | EME | Yes |  |
|  | 12/127/134 | A pragmatic randomised controlled trial of intravenous levetiracetam versus intravenous phenytoin in terminating acute, prolonged tonic–clonic seizures including convulsive status epilepticus in children, the ‘EcLiPSE’ Study: Emergency treatment with Levetiracetam or Phenytoin in Status Epilepticus. | HTA | Yes |  |
|  | 12/10/04 | A randomized controlled trial of a duodenal sleeve bypass device (Endobarrier) compared with standard medical therapy for the management of obese subjects with type 2 diabetes | EME | Yes |  |
|  | 12/167/02 | PREVenting infection using Antibiotic Impregnated Long lines (PREVAIL) | HTA | Yes |  |
|  | 12/170/45 | A randomised parallel group double blind placebo-controlled dose ranging Trial of low dose adjunctive alTeplase during priMary PCI (T-TIME). | EME | Yes |  |
|  | 11/26/05 | RATULS: Robot Assisted Training for the Upper Limb after Stroke | HTA | Yes |  |
|  | 12/201/09 | Stratified Care for Patients with Sciatica and Suspected Sciatica in Primary Care: A randomised trial (the SCOPiC trial - SCiatica Outcomes in Primary Care) | HTA | Yes |  |
|  | 11/129/187 | Human papillomavirus infection: a randomised controlled trial of Imiquimod versus Podophyllotoxin, in combination with quadrivalent human papillomavirus vaccination in the treatment and prevention of recurrence of anogenital warts (HIPvac Trial) | HTA | Yes |  |
|  | 16/95/01 | REMEDY: Randomised Evaluation of Management of sExual DYsfunction | HTA | No | Stopped early |
|  | 12/35/15 | A randomised controlled trial to determine the clinical and cost effectiveness of thulium laser transurethral vaporesection of the prostate(ThuVARP)versus transurethral resection of the prostate(TURP)in the National Health Service(NHS). | HTA | Yes |  |
|  | 06/303/98 | PERSEPHONE - duration of trastuzumab study with chemotherapy in early breast cancer: six versus twelve months | HTA | Yes |  |
|  | 12/10/19 | IMPRESS-AF: IMproved exercise tolerance in heart failure with PReserved Ejection fraction by Spironolactone on myocardial fibrosiS in Atrial Fibrillation | EME | Yes |  |
|  | 14/49/34 | An online randomised controlled trial to evaluate the clinical and cost effectiveness of a peer supported self-management intervention for relatives of people with psychosis or bipolar disorder: Relatives Education And Coping Toolkit (REACT) | HTA | Yes |  |
|  | 12/167/26 | Effectiveness of progesterone to prevent miscarriage in women with early pregnancy bleeding: A randomised placebo-controlled trial (PRISM Trial: PRogesterone In Spontaneous Miscarriage Trial) | HTA | Yes |  |
|  | 10/71/01 | Does oral sodium bicarbonate therapy improve function and quality of life in older patients with chronic kidney disease and low-grade acidosis? A multicentre randomized placebo controlled trial | HTA | Yes |  |
|  | 10/50/42 | Prospective randomised trial evaluating the efficacy of local anaesthetic thoracoscopy and talc slurry pleurodesis (TAPPS) | HTA | Yes |  |
|  | 10/37/01 | A trial to evaluate an extended rehabilitation service for stroke patients(EXTRAS) | HTA | Yes |  |
|  | 13/04/30 | A Multicentre randomised trial to establish the effect (s) of routine administration of Fluoxetine in patients with a recent stroke (FOCUS) | HTA | Yes |  |
|  | 11/30/11 | A multi-arm phase IIb randomised, double blind clinical trial comparing the efficacy of 3 neuroprotective drugs compared to placebo in secondary progressive multiple sclerosis. | EME | Yes |  |
|  | 13/153/04 | Liver Resection Surgery Versus Thermal Ablation for Colorectal LiVer MetAstases | HTA | No | Stopped early |
|  | 12/21/01 | Clinical and cost effectiveness of aphasia computer therapy compared with usual stimulation or attention control long term post stroke (CACTUS) | HTA | Yes |  |
|  | 11/01/25 | Speed of Increasing milk Feeds Trial (SIFT) | HTA | Yes |  |
|  | 11/47/01 | Minocycline in Alzheimer's Disease Efficacy Trial: The MADE Trial | EME | Yes |  |
|  | 10/104/30 | The British Antibiotic and Silver Impregnated Catheters for ventriculoperitoneal Shunts multi-centre randomised controlled trial (The BASICS trial) | HTA | Yes |  |
|  | 11/15/13 | Evaluating the effectiveness and cost effectiveness of Dementia Care Mapping (DCM) to enable person-centred Care for people with dementia and their carers: A UK cluster randomised controlled trial in care homes (DCM EPIC trial) | HTA | Yes |  |
|  | 12/33/12 | Primary care use of a C-Reactive Protein (CRP) Point of Care Test (POCT) to help target antibiotic prescribing to patients with Acute Exacerbations of Chronic Obstructive Pulmonary Disease (AECOPD) who are most likely to benefit (The PACE Study) | HTA | Yes |  |
|  | 13/115/62 | UK Study of tendo Achilles Rehabilitation – multicentre randomised clinical trial (UK STAR) | HTA | Yes |  |
|  | 12/190/05 | Cognitive Rehabilitation for Attention and Memory in people with Multiple Sclerosis (CRAMMS) | HTA | Yes |  |
|  | 07/44/03 | FiCTION - Filling Children's Teeth: Indicated Or Not? | HTA | Yes |  |
|  | 12/29/01 | A pragmatic adaptive sequential placebo controlled randomised trial to determine the effectiveness of Glyceryl trinitrate for retained placenta (Got-it trial) | HTA | Yes |  |
|  | 10/62/03 | A multi-centre randomised controlled trial comparing the effectiveness of enhanced motivational interviewing with usual care for reducing cardiovascular risk | HTA | Yes |  |
|  | 10/90/22 | The Evaluation of blood pressure treatment stratified according to Central Aortic Systolic Pressure (CASP) in Young Hypertensive Patients - The TREAT CASP study | EME | Yes |  |
|  | 14/140/84 | Extension of Follow-Up for High Risk Stage II Patients (additional 3 years) and Stage III Patients (up to year 3 follow-up) in the SCOT study | HTA | Yes |  |
|  | 12/206/30 | Platelet Rich Plasma (PRP) in Achilles Tendon Rupture (ATR) Healing | EME | Yes |  |
|  | 08/116/69 | Tackling Early Morbidity and Mortality in Myeloma (TEAMM): A Randomised Controlled Trial Assessing the benefit of antibiotic prophylaxis and its effect on healthcare associated infections | HTA | Yes |  |
|  | 11/94/01 | A cluster randomised controlled trial of a behavioural change package to prevent hand dermatitis in nurses working in the National Health Service | HTA | Yes |  |
|  | 09/100/10 | Efficacy and mechanism of thyroxine treatment on pregnancy and neonatal outcomes in women with thyroid antibodies: A randomised, placebo-controlled, double-blind, multi-centre trial [The TABLET (Thyroid AntiBodies and LEvoThyroxine) Trial] | EME | Yes |  |
|  | 11/112/01 | Reducing Arthritis Fatigue - clinical Teams using cognitive-behavioural approaches (RAFT) | HTA | Yes |  |
|  | 13/96/07 | ANODE: prophylactic ANtibiotics for the prevention of infection following Operative DElivery | HTA | Yes |  |
|  | 12/35/23 | A multicentre randomised controlled trial comparing laparoscopic supra-cervical hysterectomy with second generation endometrial ablation for the treatment of heavy menstrual bleeding (HEALTH) | HTA | Yes |  |
|  | 11/36/33 | Pressure RElieving Support SUrfaces: a Randomised Evaluation 2 (PRESSURE 2) | HTA | Yes |  |
|  | 08/52/01 | ROLARR: Robotic versus Laparoscopic Resection for Rectal Cancer | EME | Yes |  |
|  | 10/134/06 | Protocolised trial of invasive and non-invasive weaning off ventilation | HTA | Yes |  |
|  | 14/187/01 | A blinded randomised controlled trial investigating the efficacy of morphine analgesia for procedural pain in infants. | EME | Yes |  |
|  | 13/158/48 | Minidex: The efficacy and safety of very low dose dexamethasone used to facilitate the extubation of ventilator dependent preterm babies who are at high risk of bronchopulmonary dysplasia. | EME | No | Feasibility |
|  | 09/100/23 | The Benefit of Minocycline on Negative Symptoms in Schizophrenia: Extent and Mechanisms | EME | Yes |  |
|  | 10/99/01 | Physiotherapy Rehabilitation for Osteoporotic VErtebral fracture trial (PROVE) | HTA | Yes |  |
|  | 09/144/50 | Randomised controlled trial of the clinical and cost-effectiveness of a contingency management intervention for reduction of cannabis use and of relapse in early psychosis | HTA | Yes |  |
|  | 12/167/135 | A randomised controlled trial to examine the efficacy of e-cigarettes compared with nicotine replacement therapy, when used within the UK stop smoking service | HTA | Yes |  |
|  | 11/129/16 | Does vertebral stenting prevent recurrent stroke in symptomatic vertebral artery stenosis: The Vertebral artery Ischaemia Stenting Trial (VIST) | HTA | Yes |  |
|  | 11/143/01 | Development and validation of the 4AT: a new rapid screening tool for delirium | HTA | No | Not an RCT |
|  | 11/36/29 | Oral Versus Intravenous Antibiotics (OVIVA) for Bone and Joint Infection | HTA | Yes |  |
|  | 11/58/15 | A randomised, double-blind placebo controlled trial of the effectiveness of low dose oral theophylline as an adjunct to inhaled corticosteroids in preventing exacerbations of chronic obstructive pulmonary disease. | HTA | Yes |  |
|  | 10/57/21 | A Randomised Controlled Trial of the Effectiveness of PDSAFE to prevent Falls among People with Parkinson's Disease | HTA | Yes |  |
|  | 11/129/109 | Tranexamic acid for hyperacute primary Intracerebral Haemorrhage (TICH-2) | HTA | Yes |  |
|  | 10/60/37 | TAILoR (TelmisArtan and InsuLin Resistance in HIV): A Dose-Ranging Phase II Randomised Open-Labelled Trial of Telmisartan as a Strategy for the Reduction of Insulin Resistance in HIV-Positive Individuals on Combination Antiretroviral Therapy (cART) | EME | Yes |  |
|  | 13/88/13 | Children’s drops for ear pain in acute otitis media: the CEDAR randomised controlled trial | HTA | Yes |  |
|  | 08/43/61 | Does Co-careldopa treatment in combination with routine NHS occupational and physical therapy, delivered early after stroke within a stroke rehabilitation service, improve functional recovery including walking ability and arm function? | EME | Yes |  |
|  | 09/100/25 | A randomised controlled trial of eicosapentaenoic acid (EPA) and/or aspirin for colorectal adenoma (or polyp) prevention during colonoscopic surveillance in the NHS Bowel Cancer Screening Programme: The seAFOod polyp prevention trial | EME | Yes |  |
|  | 12/33/28 | A multi-centre, randomised, double blind, placebo-controlled, parallel group trial of the effectiveness of the nocturnal use of a Temperature Controlled Laminar Airflow (TLA) Device (Airsonett®) in adults with poorly-controlled, severe allergic asthma: the LASER trial | HTA | Yes |  |
|  | 08/53/31 | Long-term tapering versus standard prednisolone (steroid) therapy for the treatment of the initial episode of childhood nephrotic syndrome: national multicentre randomised double blind controlled trial | HTA | Yes |  |
|  | 07/89/01 | FIAT (Fistula-in-ano trial) comparing Surgisis® anal fistula plug versus surgeon's preference (advancement flap, fistulotomy, cutting seton) for transsphincteric fistula-in-ano | HTA | Yes |  |
|  | 12/127/10 | A randomised controlled trial to compare the clinical effectiveness and safety of gentamicin and ceftriaxone in the treatment of gonorrhoea | HTA | Yes |  |
|  | 09/33/02 | CanTalk: the clinical and cost effectiveness of CBT plus treatment as usual for the treatment of depression in advanced cancer; a randomised controlled trial | HTA | Yes |  |
|  | 10/57/24 | Rehabilitation of Memory following Traumatic Brain Injury - a Phase III Randomised Controlled Trial | HTA | Yes |  |
|  | 09/51/01 | Phase III randomised controlled trial of the clinical effectiveness, safety and cost effectiveness of adalimumab for the treatment of juvenile idiopathic arthritis associated uveitis | HTA | Yes |  |
|  | 13/88/10 | Electronically-delivered, multi-component interventions to reduce unnecessary antibiotic prescribing in primary care. A cluster randomised trial using electronic health records (eCRT2) | HTA | Yes |  |
|  | 11/15/04 | Goal-oriented cognitive rehabilitation in early-stage Alzheimer's disease: multi-centre single-blind randomised controlled trial (GREAT) | HTA | Yes |  |
|  | 11/30/02 | A multicentre phase III randomised controlled single masked clinical trial to test the clinical efficacy of LightMasks at preventing dark adaptation in the treatment of early diabetic macular oedema (CLEOPATRA) | EME | Yes |  |
|  | 10/101/02 | Focusing On Clozapine Unresponsive Symptoms (FOCUS): a randomised controlled trial | HTA | Yes |  |
|  | 11/14/34 | Selection of sperm for Assisted Reproductive Treatment by prior hyaluronic acid binding: increasing live birth outcomes and reducing miscarriage rates (Hyaluronic Acid Binding Sperm Selection; HABSelect). | EME | Yes |  |
|  | 10/46/01 | Naltrexone Enhanced Addiction Treatment (NEAT) for opioid dependence. A randomised controlled trial of the clinical and cost-effectiveness of implanted extended-release naltrexone and oral naltrexone | HTA | No | Stopped early |
|  | 10/57/20 | A randomised controlled trial of standard-of-care wound management versus negative pressure wound therapy in the treatment of adult patients with an open fracture of the lower limb | HTA | Yes |  |
|  | 10/57/14 | A multi-centre randomised placebo-controlled trial of prophylactic enteral supplementation with bovine lactoferrin to prevent late-onset invasive infection in very preterm or very low birth weight infants. | HTA | Yes |  |
|  | 09/150/12 | Radically Open Dialectical Behaviour Therapy for Treatment-Resistant Depression: A Randomised Controlled Trial | EME | Yes |  |
|  | 10/143/01 | The clinical and cost-effectiveness of temporarily quadrupling the dose of inhaled steroid to prevent asthma exacerbations; a pragmatic, randomised, normal care-controlled, clinical trial | HTA | Yes |  |
|  | 09/55/06 | A practical randomised controlled double-blind trial of antipsychotic treatment of very late-onset schizophrenia-like psychosis: The ATLAS Trial | HTA | Yes |  |
|  | 12/28/05 | STEPWISE: STructured lifestyle Education for People WIth Schizophrenia | HTA | Yes |  |
|  | 11/129/76 | A double blind placebo-controlled randomised trial of the addition of the antidepressant mirtazapine for patients with depression in primary care who have not responded to at least 6 weeks of antidepressant treatment | HTA | Yes |  |
|  | 11/14/08 | An efficacy and mechanism evaluation study of Levosimendan for the Prevention of Acute oRgan Dysfunction in Sepsis (LeoPARDS) | EME | Yes |  |
|  | 11/01/26 | Oral STeroids for Resolution of otitis media with effusion (OME) In CHildren: the OSTRICH study | HTA | Yes |  |
|  | 10/104/25 | Adjunctive Rifampicin to Reduce Early mortality from Staphylococcus aureus bacteraemia: a multi-centre, randomised, double blind, placebo controlled trial (the ARREST trial) | HTA | Yes |  |
|  | 12/127/12 | Abdominal massage for neurogenic bowel dysfunction in people with multiple sclerosis (AMBER Abdominal Massage for Bowel Dysfunction Effectiveness Research) | HTA | Yes |  |
|  | 11/153/01 | BATHE (Bath Emollients for Treatment of cHildhood Eczema) | HTA | Yes |  |
|  | 12/66/15 | Clinical Efficacy and Mechanistic Evaluation of Aflibercept for Proliferative Diabetic Retinopathy (acronym CLARITY) | EME | Yes |  |
|  | 10/104/34 | BEAT-IT: A randomised controlled trial comparing a behavioural activation treatment for depression in adults with learning disabilities with an attention control. | HTA | Yes |  |
|  | 10/104/24 | Safety and efficacy of Triple Antiplatelets for Reducing Dependency after Ischaemic Stroke: the TARDIS randomised controlled trial | HTA | Yes |  |
|  | 11/01/30 | European study of therapeutic hypothermia (32-35°C) for intracranial pressure reduction after traumatic brain injury (Eurotherm3235) | HTA | Yes |  |
|  | 08/14/39 | Randomised controlled trial of continuous subcutaneous insulin infusion compared to multiple daily injection regimens in children and young people at diagnosis of type I diabetes mellitus | HTA | Yes |  |
|  | 09/110/01 | A randomised trial of nicotine patch preloading for smoking cessation | HTA | Yes |  |
|  | 09/22/50 | Can magnetic resonance imaging scan replace, or triage the use of laparoscopy in establishing diagnosis among women presenting in secondary care with chronic pelvic pain? | HTA | No | Not an RCT |
|  | 09/01/45 | Improving the Quality of Dentistry (IQuaD): A randomised controlled trial comparing oral hygiene advice and periodontal instrumentation for the prevention and management of periodontal disease in dentate adults attending dental primary care | HTA | Yes |  |
|  | 10/32/02 | Randomised controlled trial of a pedometer-based walking intervention with and without practice nurse support in primary care patients aged 45-74 years | HTA | Yes |  |
|  | 10/60/30 | Clinical efficacy of functional strength training for upper limb motor recovery early after stroke: neural correlates and prognostic indicators | EME | Yes |  |
|  | 08/116/75 | SWITCH - Randomised- controlled trial of switching to alternative tumour necrosis factor-blocking drugs or abatacept or rituximab in patients with rheumatoid arthritis who have failed an initial TNF-blocking drug | HTA | Yes |  |
|  | 07/37/64 | Can emergency endovascular aneurysm repair (eEVAR) improve the survival from ruptured abdominal aortic aneurysm? | HTA | Yes |  |
|  | 09/80/04 | Physical activity programmes for community dwelling people with mild to moderate dementia (DAPA - Dementia And Physical Activity) | HTA | Yes |  |
|  | 11/136/04 | For patients with a displaced fracture of the distal tibia, is there a clinical and cost-effectiveness difference between ‘locking’ plate fixation and intramedullary nail fixation? (FixDT) | HTA | Yes |  |
|  | 11/72/01 | Antibiotic treatment for intermittent bladder catheterisation: A randomised controlled trial of once daily prophylaxis (The AnTIC study) | HTA | Yes |  |
|  | 09/55/38 | Antiepileptic drug (AED) management in Pregnancy: An evaluation of effectiveness, cost effectiveness and acceptability of dose adjustment strategies | HTA | Yes |  |
|  | 10/90/03 | Rituximab for the Treatment of Fatigue in Primary Biliary Cirrhosis | EME | Yes |  |
|  | 09/165/01 | Self-Management education for adults with poorly controlled epILEpsy (SMILE) A Randomised Controlled Trial | HTA | Yes |  |
|  | 07/01/34 | A randomised controlled trial to investigate the clinical and cost effectiveness of adding an ablation device-based maze procedure as a routine adjunct to elective cardiac surgery for patients with pre-existing atrial fibrillation (AMAZE) | HTA | Yes |  |
|  | 10/103/01 | The clinical and cost effectiveness of lamotrigine for people with borderline personality disorder: Randomised controlled trial | HTA | Yes |  |
|  | 10/104/13 | Clinical and cost effectiveness of staff training in Positive Behaviour Support (PBS) for treating challenging behaviour among people with learning disability: a multicentre cluster randomised controlled trial | HTA | Yes |  |
|  | 09/104/21 | The Stroke Oxygen Study: a multi-centre, prospective, randomised, open, blinded-endpoint study to assess whether routine oxygen treatment in the first 72 hours after a stroke improves long-term outcome | HTA | Yes |  |
|  | 10/104/16 | Improving outcomes in adults with epilepsy and intellectual disability: A cluster randomised controlled trial of nurse-led epilepsy management (EpAID) | HTA | Yes |  |
|  | 06/85/11 | A cluster randomised controlled trial of the effectiveness and cost-effectiveness of an obesity prevention intervention in primary school age children | HTA | Yes |  |
|  | 06/38/01 | A multicentre randomised controlled trial of an intelligent system to support decision making in the management of labour using the cardiotocogram (INFANT) | HTA | Yes |  |
|  | 08/29/02 | PET-PANC | HTA | No | Not an RCT |
|  | 08/99/08 | Hydroxymethylglutaryl-CoA reductase inhibition with simvastatin in Acute lung injury to Reduce Pulmonary dysfunction (HARP-2) | EME | Yes |  |
|  | 10/57/32 | A randomised controlled trial of intra-operative cell salvage during caesarean section in women at risk of haemorrhage. | HTA | Yes |  |
|  | 08/24/02 | A pragmatic multicentre randomised controlled trial comparing stapled haemorrhoidopexy to conventional excisional surgery for haemorrhoidal disease. | HTA | Yes |  |
|  | 10/57/43 | The CASPER-PLUS Trial: Collaborative care for screen-positive elders with Major Depressive Disorder | HTA | Yes |  |
|  | 11/14/33 | Low-dose Intravenous Immunoglobulin Treatment for Complex Regional Pain Syndrome (‘LIPS’) Randomised Controlled Trial. | EME | Yes |  |
|  | 08/22/02 | A study of position during the late stages of labour in women with an epidural (BUMPES) | HTA | Yes |  |
|  | 11/146/01 | Early versus delayed surgery for congenital hip dysplasia | HTA | No | Stopped early |
|  | 09/144/51 | The Age of Blood Evaluation Study (ABLE) | HTA | Yes |  |
|  | 12/201/02 | A randomised controlled trial of adalimumab injection compared with placebo injection for patients receiving physiotherapy treatment for sciatica.  Trial acronym: Sub-Cutaneous Injection of Adalimumab Trial compared with Control (SCIATiC) | HTA | No | Stopped early |
|  | 12/167/95 | Randomised controlled trial of improvisational music therapy for children with autism spectrum disorders: UK arm of the TIME-A study | HTA | Yes |  |
|  | 06/301/233 | Asymptomatic Carotid Surgery Trial-2 (ACST-2): an international randomised trial to compare carotid endarterectomy with carotid artery stenting to prevent stroke | HTA | No | Trial ongoing |
|  | 12/01/16 | A very early rehabilitation trial after stroke (AVERT): A phase 3, multicentre, randomised controlled trial | HTA | Yes |  |
|  | 09/104/19 | A controlled study of the effectiveness of breathing training exercises taught by a physiotherapist by either instructional videos/DVDs/internet download or by face-to-face sessions in the management of asthma in adults | HTA | Yes |  |
|  | 08/116/12 | Amisulpride augmentation in clozapine-unresponsive schizophrenia AMICUS | HTA | Yes |  |
|  | 10/104/20 | Community led ANti-psychotic Drug REduction for Adults with Learning Disabilities (ANDREA-LD): A Randomised Double-blind Placebo Controlled Trial | HTA | No | Stopped early |
|  | 10/50/14 | COBRA (Cost and Outcome of BehaviouRal Activation): a Randomised Controlled Trial of Behavioural Activation versus Cognitive Behaviour Therapy for Depression | HTA | Yes |  |
|  | 13/28/02 | Randomised controlled trial of the selective serotonin reuptake inhibitor Sertraline versus Cognitive Behavioural Therapy for anxiety symptoms in people with Generalised Anxiety Disorder who have failed to respond to low intensity psychological interventions as defined by the NICE GAD guidelines | HTA | No | Pilot |
|  | 09/81/01 | Obsessive Compulsive Treatment Efficacy Trial (OCTET) | HTA | Yes |  |
|  | 11/148/01 | DeCoDer Trial Debt Counselling for Depression in Primary Care: an Adaptive Randomised Controlled Trial. | HTA | No | Pilot |
|  | 09/01/53 | Evaluation of lightweight fibreglass heel casts in the management of ulcers of the heel in diabetes | HTA | Yes |  |
|  | 99/10/99 | A randomised controlled trial to assess the cost-effectiveness of intensive versus no scheduled follow-up in patients who have undergone resection for colorectal cancer with curative intent (The FACS Trial) | HTA | Yes |  |
|  | 11/129/148 | A multicentre randomiSed controlled TRial of IntraVEnous Immunoglobulin (IVIg) versus standard therapy for the treatment of transverse myelitis in adults and children (STRIVE trial) | HTA | No | Stopped early |
|  | 11/31/01 | Facet Feasibility (FF) | HTA | No | Feasibility |
|  | 07/01/38 | ARCTIC: A randomised, phase IIB trial in previously untreated patients with Chronic Lymphocytic Leukaemia (CLL) to compare fludarabine, cyclophosphamide and rituximab (FCR) with FC, mitoxantrone and low dose rituximab (FCM-miniR) | HTA | Yes |  |
|  | 09/77/01 | Randomised trial of a multifaceted podiatry intervention for fall prevention | HTA | Yes |  |
|  | 08/104/04 | Seal or Varnish? A randomised trial to determine the relative cost and effectiveness of pit and fissure sealants and fluoride varnish in preventing dental decay | HTA | Yes |  |
|  | 08/107/01 | The REPOSE (Relative Effectiveness of Pumps over MDI and Structured Education) Trial | HTA | Yes |  |
|  | 09/22/163 | Development and validation of a Prediction model for Risk of complications in Early onset Pre eclampsia (PREP) | HTA | No | Not an RCT |
|  | 06/302/129 | Positron Emission Tomography-Computerised Tomography scans (PET-CT) guided watch and wait policy versus planned neck dissection for the management of locally advanced (N2/N3) nodal metastases in patients with head and neck squamous cancer | HTA | Yes |  |
|  | 11/65/01 | Randomised controlled trial of silk therapeutic clothing for the long-term management of eczema in children (CLOTHES Trial: CLOTHing for the relief of Eczema Symptoms) | HTA | Yes |  |
|  | 07/37/69 | Prehospital Randomised Assessment of a Mechanical Compression Device in out of Hospital Cardiac Arrest (PARAMEDIC): a pragmatic cluster randomised trial and economic evaluation | HTA | Yes |  |
|  | 07/01/21 | Care of older people who fall: evaluation of the clinical and cost effectiveness of new protocols for emergency ambulance paramedics to assess and refer to appropriate community based care | HTA | Yes |  |
|  | 06/05/01 | Effectiveness And Cost Effectiveness Of Cognitive Behaviour Therapy And Short-Term Psychoanalytic Psychotherapy Compared With Brief Psychosocial Intervention In The Maintenance Of Symptomatic Remission In Adolescents with Unipolar Major Depression (IMPACT): A Randomised Controlled Trial | HTA | Yes |  |
|  | 10/90/04 | Ketamine augmentation of ECT to improve outcomes in depression | EME | Yes |  |
|  | 06/403/51 | A randomised controlled trial to compare the safety and effectiveness of doxycycline (200 mg/day) with prednisolone (0.5 mg/kg/day) for initial treatment of bullous pemphigoid | HTA | Yes |  |
|  | 08/19/04 | Collaborative Care in Screen-Positive Elders – The CASPER Trial | HTA | Yes |  |
|  | 09/127/19 | POWER STUDY - Positive Online Weight Reduction | HTA | Yes |  |
|  | 08/58/02 | A randomised trial to increase the uptake of smoking cessation services using Personal Targeted risk information and Taster Sessions | HTA | Yes |  |
|  | 09/127/41 | A randomised controlled trial evaluating the effectiveness and cost-effectiveness of ‘Families for Health’, a family-based childhood obesity treatment intervention delivered in a community setting for ages 6 to 11 | HTA | Yes |  |
|  | 11/01/10 | PLEASANT: Preventing and Lessening Exacerbations of Asthma in School-age children Associated with a New Term | HTA | Yes |  |
|  | 11/20/05 | A Randomised, Double-blind, Single-centre, Controlled Trial of Low Dose Intradermal Allergen Immunotherapy in Adults with Seasonal Allergic Rhinitis | EME | Yes |  |
|  | 09/150/28 | Randomized Controlled Trial Comparing Intracoronary Administration of Adenosine or Sodium Nitroprusside to Control for Attenuation of Microvascular Obstruction During Primary Percutaneous Coronary Intervention | EME | Yes |  |
|  | 08/64/01 | The role of ultrasound compared to biopsy of temporal arteries in the diagnosis and treatment of giant cell arteritis | HTA | No | Not an RCT |
|  | 10/57/46 | A multi-centre randomised controlled trial comparing rubber band ligation with haemorrhoidal artery ligation in the management of symptomatic second and third degree haemorrhoids | HTA | Yes |  |
|  | 06/43/504 | The Randomised Evaluation of the Effectiveness and Acceptability of Computerised Therapy (REEACT) Trial | HTA | Yes |  |
|  | 10/82/01 | A Study of Donor Ex-vivo Lung Perfusion in UK Lung Transplantation: DEVELOP-UK | HTA | No | Not an RCT |
|  | 11/129/61 | Enhanced Invitation Methods to Increase Uptake of NHS Health Checks. Randomised Controlled Trial | HTA | Yes |  |
|  | 09/127/34 | A peer-support weight action programme to supplement brief advice in general practice | HTA | Yes |  |
|  | 10/60/27 | A Randomised, Double-Blind, Placebo-Controlled Study to Evaluate the Efficacy of Oral Azithromycin (500mg OD) as a Supplement to Standard Care for Adult Patients with Acute Exacerbations of Asthma (the AZALEA Trial). | EME | Yes |  |
|  | 07/37/61 | AIM: Ankle Injury Management - A pragmatic multi-centre randomised controlled trial comparing close contact casting technique (CCC) to open surgical reduction and internal fixation (ORIF) in the treatment of unstable ankle fractures in patients over 60 years | HTA | Yes |  |
|  | 07/60/49 | An international randomised controlled trial to compare targeted intra-operative radiotherapy (TARGIT) with conventional post-operative radiotherapy for women with early breast cancer. | HTA | Yes |  |
|  | 08/14/19 | A randomised controlled trial to measure the effects and costs of a dental caries prevention regime for young children attending primary care dental services | HTA | Yes |  |
|  | 07/60/43 | Financial incentives to improve adherence to anti-psychotic maintenance medication in non-adherent patients - a cluster randomised controlled trial: FIAT | HTA | Yes |  |
|  | 09/164/01 | Strategies to increase cervical screening uptake at first invitation (STRATEGIC) | HTA | Yes |  |
|  | 08/246/09 | Efficacy of Metformin in Pregnant Obese Women, a Randomised Controlled Trial. | EME | Yes |  |
|  | 08/116/300 | Anglo-Danish-Dutch study of Intensive Treatment of people with Newly diagnosed diabetes in primary care (ADDITION)–five year follow-up | HTA | Yes |  |
|  | 07/01/07 | Randomised Controlled Trial to assess the clinical- and cost-effectiveness of Physiotherapy and Occupational Therapy in Parkinson's Disease (PD REHAB) | HTA | Yes |  |
|  | 05/501/04 | PREFER: Trial of probiotic administered early to prevent infection and necrotising enterocolitis | HTA | Yes |  |
|  | 11/100/24 | Next Generation intraoperative Lymph node staging for Stratified colon cancer surgery (GLiSten) | EME | No | Feasibility |
|  | 06/402/94 | A multi-centre randomised controlled trial of Transfusion Indication Threshold Reduction on transfusion rates, morbidity and healthcare resource use following cardiac surgery (TITRe 2) | HTA | Yes |  |
|  | 09/70/04 | Strategies To incRease confidence, InDependence and Energy - Cognitive behavioural therapy-based intervention to reduce fear of falling in older patients attending a community falls service: Therapy development and randomised controlled trial. | HTA | Yes |  |
|  | 08/53/06 | Psycho-education with problem solving (PEPS) therapy for adults with personality disorder: A community-based randomised controlled trial | HTA | Yes |  |
|  | 06/303/205 | A randomised phase III trial of Docetaxel plus Prednisolone vs. Docetaxel with Prednisolone plus either Zoledronic acid, Strontium-89 or both agents combined (TRAPEZE) | HTA | Yes |  |
|  | 11/14/25 | A randomised double-blind placebo controlled Phase 2B clinical trial of repeated application of gene therapy in patients with Cystic Fibrosis | EME | Yes |  |
|  | 08/53/25 | BREATHER (PENTA 16) Short-Cycle Therapy (SCT) (5 days on/2 days off) in young people with chronic HIV-infection | HTA | Yes |  |
|  | 09/55/33 | DiPALS: A randomised controlled trial evaluating NeuRx/4 Diaphragm Pacing in patients with respiratory muscle weakness due to Motor Neurone Disease | HTA | Yes |  |
|  | 09/100/05 | Effect of Remote Ischaemic preConditioning on Clinical outcomes in patients undergoing Coronary Artery bypass graft surgery (ERICCA study): A multicentre double-blind randomised controlled clinical trial | EME | Yes |  |
|  | 06/78/03 | Comparison Of iNfliximab and ciclosporin in STeroid Resistant Ulcerative Colitis: a Trial (CONSTRUCT) | HTA | Yes |  |
|  | 08/38/01 | First trimester progesterone therapy in women with a history of unexplained recurrent miscarriages: A randomised, double-blind, placebo-controlled, multi-centre trial [The PROMISE (PROgesterone in recurrent MIScarriage) Trial] | HTA | Yes |  |
|  | 07/83/01 | ANTIDEPRESSANT CONTROLLED TRIAL FOR NEGATIVE SYMPTOMS IN SCHIZOPHRENIA (ACTIONS) A double-blind, placebo-controlled, randomised clinical trial | HTA | Yes |  |
|  | 07/52/03 | CALORIES: A phase III, open, multicentre, randomised controlled trial comparing the clinical and cost-effectiveness of early nutritional support in critically ill patients via the parenteral versus the enteral route | HTA | Yes |  |
|  | 08/99/24 | WT1 Immunity via DNA fusion Gene Vaccination in Haematological Malignancies by intramuscular injection followed by intramuscular electroporation. | EME | No | Not an RCT |
|  | 08/99/04 | Amino acid regimen and intravenous lipid composition in preterm parenteral nutrition: a randomised double blind controlled trial of Nutritional Evaluation and Optimisation in Neonates (NEON) | EME | Yes |  |
|  | 06/403/90 | A randomised controlled trial of a strategy of switching to boosted protease inhibitor monotherapy versus continuing combination antiretroviral therapy for the long-term management of HIV-1 infected patients who have achieved sustained virological suppression on highly-active antiretroviral therapy | HTA | Yes |  |
|  | 09/118/03 | The CREAM Study - Children With Eczema, Antibiotic Management study | HTA | Yes |  |
|  | 08/13/47 | CATheter Infections in Children - the CATCH trial | HTA | Yes |  |
|  | 08/14/30 | A cluster randomised controlled trial of an occupational therapy intervention for residents with stroke living in UK care-homes | HTA | Yes |  |
|  | 08/116/68 | Effectiveness and cost-effectiveness of body psychotherapy in the treatment of negative symptoms of schizophrenia: a multicentre randomised controlled trial | HTA | Yes |  |
|  | 10/27/01 | The randomised Complete versus Lesion-only PRimary percutaneous coronary Intervention Trial: Cardiovascular Magnetic Resonance imaging substudy (CvLPRIT-CMR) | EME | Yes |  |
|  | 09/22/122 | Accuracy of Bladder ultrasound (BUS) in the diagnosis of Detrusor Overactivity (DO): a study to evaluate if ultrasound can reduce the need for urodynamics | HTA | No | Not an RCT |
