# Supplementary material for: Developing guidance for a risk-proportionate approach to blinding statisticians within clinical trials: a mixed methods study
Source: Trials. 2023 Jan 31;24:71. doi: 10.1186/s13063-022-06992-5 (PMC9887916; doi:10.1186/s13063-022-06992-5)
Supplement: Supplementary file 4 — Additional file 4. Additional analyses. [file 13063_2022_6992_MOESM4_ESM.docx]

**Additional file 4 – Additional analyses**

**Table A1: Sensitivity analysis - Odds ratios (OR) describing the influence of the blinding status of the statistician and other selected study design features on whether a statistically significant finding is reported**

| **Blinding status of the statistician known** | | |  |
| --- | --- | --- | --- |
| N = 152 | **OR (95% CI)^1^** | **p-value** |  |
| Statistician not blinded | 0.90 (0.42, 1.92) | 0.780 |  |
| Multiple comparisons^2^ | 1.32 (0.61, 2.87) | 0.485 |  |
| Target sample size achieved | 0.99 (0.43, 2.31) | 0.987 |  |
| Participants not blinded^3^ | 0.63 (0.15, 2.61) | 0.522 |  |
| Clinicians not blinded^3^ | 4.09 (0.86, 19.37) | 0.076 |  |
| Outcome assessors not blinded^3^ | 2.00 (0.88, 4.52) | 0.096 |  |
| **Imputation of missing blinding status of the statistician^4^** | | |  |
| N = 179 | **OR (95% CI)^1^** | **p-value** |  |
| Statistician not blinded | 0.98 (0.48, 1.97) | 0.944 |  |
| Multiple comparisons^2^ | 1.44 (0.71, 2.93) | 0.314 |  |
| Sample size achieved | 1.29 (0.60, 2.77) | 0.515 |  |
| Participants not blinded^3^ | 1.23 (0.33, 4.55) | 0.758 |  |
| Clinicians not blinded^3^ | 1.77 (0.44, 7.13) | 0.420 |  |
| Outcome assessors not blinded^3^ | 1.91 (0.91, 4.02) | 0.086 |  |
| ^1^Odds ratio (and 95% confidence interval) from logistic regression model comparing the influence of the listed study design features on the likelihood of a statistically significant finding.  ^2^A trial is defined as having multiple comparisons if it contains more than two treatment groups or multiple co-primary outcomes. ^3^To facilitate inclusion in the model where the blinding status is unclear this was assumed as not blinded.  ^4^Where blinding status is unclear it was assumed the statistician was not blinded. | | | |

**Table A2: Secondary analysis – Statistical models describing the influence of the blinding status of the statistician and other selected study design features on the reported p-value**

| **Blinding status of the statistician known** | | |  |
| --- | --- | --- | --- |
| N = 95 | **Coefficient (95% CI)^1^** | **p-value** |  |
| Statistician not blinded | 0.14 (−0.32, 0.61) | 0.545 |  |
| Target sample size achieved | 0.36 (−0.19, 0.91) | 0.199 |  |
| Trial not blinded^2^ | −0.31 (−0.83, 0.20) | 0.234 |  |
| **Imputation of missing blinding status of the statistician^3^** | | |  |
| N = 110 | **Coefficient (95% CI)^1^** | **p-value** |  |
| Statistician not blinded | 0.12 (−0.32, 0.57) | 0.580 |  |
| Target sample size achieved | 0.32 (−0.17, 0.81) | 0.195 |  |
| Trial not blinded^2^ | −0.22 (−0.70, 0.27) | 0.377 |  |
| ^1^Coefficient (and 95% confidence interval) from a beta regression model with logit link function and log link function for the conditional mean and conditional scale, respectively.  ^2^A trial is defined as not blinded if any of the participants, clinicians, and outcome assessors are not blinded. To facilitate inclusion in the model wherever the blinding status is unclear this was assumed as not blinded.  ^3^Where blinding status is unclear it was assumed the statistician was not blinded. | | | |

**Further exploratory analyses to check sensitivity of primary analysis findings to alternative model specification**

**Including percentage of missing data in the primary model**

No imputation on blinding status of statistician (N = 148)

| Significant finding reported | Odds ratio | Std. err. | z | P>\|z\| | [95% conf. interval] | | |
| --- | --- | --- | --- | --- | --- | --- | --- |
| **Statistician not blinded** | **0.82** | **0.33** | **-0.49** | **0.622** | **0.37** | **1.81** |  |
| Multiple comparisons | 1.56 | 0.64 | 1.08 | 0.282 | 0.69 | 3.50 |  |
| Sample size achieved | 1.20 | 0.52 | 0.42 | 0.671 | 0.51 | 2.81 |  |
| Trial not blinded | 3.25 | 1.63 | 2.35 | 0.019 | 1.22 | 8.69 |  |
| Percentage of missing data | 0.95 | 0.02 | -2.35 | 0.019 | 0.91 | 0.99 |  |
| Constant | 0.22 | 0.14 | -2.41 | 0.016 | 0.06 | 0.76 |  |

Sensitivity analysis - assuming that where the blinding status was unclear or no-response from authors the statistician was not blinded (N = 175).

| Significant finding reported | Odds ratio | Std. err. | z | P>\|z\| | [95% conf. interval] | | |
| --- | --- | --- | --- | --- | --- | --- | --- |
| **Statistician not blinded** | **0.91** | **0.34** | **-0.25** | **0.802** | **0.44** | **1.89** |  |
| Multiple comparisons | 1.55 | 0.58 | 1.17 | 0.241 | 0.75 | 3.23 |  |
| Sample size achieved | 1.48 | 0.58 | 1.00 | 0.319 | 0.69 | 3.19 |  |
| Trial not blinded | 2.92 | 1.36 | 2.31 | 0.021 | 1.18 | 7.26 |  |
| Percentage of missing data | 0.96 | 0.02 | -2.32 | 0.020 | 0.92 | 0.99 |  |
| Constant | 0.19 | 0.11 | -2.84 | 0.005 | 0.06 | 0.59 |  |

**Including percentage of missing data in the secondary model**

No imputation on blinding status of statistician (N = 148)

| Significant finding reported | Odds ratio | Std. err. | z | P>\|z\| | [95% conf. interval] | |
| --- | --- | --- | --- | --- | --- | --- |
| **Statistician not blinded** | **0.74** | **0.31** | **-0.72** | **0.470** | **0.33** | **1.67** |
| Multiple comparisons | 1.57 | 0.66 | 1.07 | 0.286 | 0.69 | 3.59 |
| Sample size achieved | 1.02 | 0.45 | 0.04 | 0.969 | 0.43 | 2.42 |
| Participants not blinded | 0.86 | 0.63 | -0.20 | 0.840 | 0.20 | 3.64 |
| Clinicians not blinded | 3.89 | 3.07 | 1.72 | 0.086 | 0.83 | 18.30 |
| Outcome assessor not blinded | 1.60 | 0.69 | 1.09 | 0.277 | 0.68 | 3.74 |
| Percentage of missing data | 0.95 | 0.02 | -2.31 | 0.021 | 0.91 | 0.99 |
| Constant | 0.22 | 0.13 | -2.52 | 0.012 | 0.07 | 0.71 |

Sensitivity analysis - assuming that where the blinding status was unclear or no-response from authors the statistician was not blinded (N = 175).

| Significant finding reported | Odds ratio | Std. err. | z | P>\|z\| | [95% conf. interval] | |
| --- | --- | --- | --- | --- | --- | --- |
| **Statistician not blinded** | **0.85** | **0.32** | **-0.42** | **0.675** | **0.41** | **1.79** |
| Multiple comparisons | 1.64 | 0.62 | 1.29 | 0.195 | 0.78 | 3.46 |
| Sample size achieved | 1.37 | 0.54 | 0.78 | 0.434 | 0.63 | 2.99 |
| Participants not blinded | 1.55 | 1.06 | 0.63 | 0.526 | 0.40 | 5.96 |
| Clinicians not blinded | 1.69 | 1.21 | 0.73 | 0.464 | 0.41 | 6.89 |
| Outcome assessor not blinded | 1.62 | 0.63 | 1.24 | 0.216 | 0.75 | 3.47 |
| Percentage of missing data | 0.96 | 0.02 | -2.28 | 0.023 | 0.92 | 0.99 |
| Constant | 0.19 | 0.11 | -2.94 | 0.003 | 0.06 | 0.58 |

**Backwards elimination of key variables**

No imputation on blinding status of statistician (N = 148)

| Significant finding reported | Odds ratio | Std. err. | z | P>\|z\| | [95% conf. interval] | |
| --- | --- | --- | --- | --- | --- | --- |
| **Statistician not blinded** | **0.82** | **0.33** | **-0.50** | **0.619** | **0.37** | **1.80** |
| Clinicians not blinded | 4.26 | 2.13 | 2.90 | 0.004 | 1.60 | 11.38 |
| Percentage of missing data | 0.95 | 0.02 | -2.37 | 0.018 | 0.91 | 0.99 |
| Constant | 0.25 | 0.13 | -2.68 | 0.007 | 0.09 | 0.69 |

Sensitivity analysis - assuming that where the blinding status was unclear or no-response from authors the statistician was not blinded (N = 175).

| Significant finding reported | Odds ratio | Std. err. | z | P>\|z\| | [95% conf. interval] | |
| --- | --- | --- | --- | --- | --- | --- |
| **Statistician not blinded** | **0.89** | **0.33** | **-0.32** | **0.747** | **0.43** | **1.82** |
| Clinicians not blinded | 2.98 | 1.31 | 2.49 | 0.013 | 1.26 | 7.06 |
| Percentage of missing data | 0.96 | 0.02 | -2.22 | 0.027 | 0.93 | 1.00 |
| Constant | 0.28 | 0.13 | -2.67 | 0.008 | 0.11 | 0.71 |

**Forwards selection of key variables**

No imputation on blinding status of statistician (N = 148)

| Significant finding reported | Odds ratio | Std. err. | z | P>\|z\| | [95% conf. interval] | |
| --- | --- | --- | --- | --- | --- | --- |
| **Statistician not blinded** | **0.82** | **0.33** | **-0.50** | **0.619** | **0.37** | **1.80** |
| Clinicians not blinded | 4.26 | 2.13 | 2.90 | 0.004 | 1.60 | 11.38 |
| Percentage of missing data | 0.95 | 0.02 | -2.37 | 0.018 | 0.91 | 0.99 |
| Constant | 0.25 | 0.13 | -2.68 | 0.007 | 0.09 | 0.69 |

Sensitivity analysis - assuming that where the blinding status was unclear or no-response from authors the statistician was not blinded (N = 175).

| Significant finding reported | Odds ratio | Std. err. | z | P>\|z\| | [95% conf. interval] | |
| --- | --- | --- | --- | --- | --- | --- |
| **Statistician not blinded** | **0.86** | **0.32** | **-0.41** | **0.684** | **0.42** | **1.78** |
| Outcome assessor not blinded | 1.71 | 0.65 | 1.41 | 0.159 | 0.81 | 3.58 |
| Percentage of missing data | 0.96 | 0.02 | -2.11 | 0.035 | 0.93 | 1.00 |
| Clinicians not blinded | 2.38 | 1.12 | 1.84 | 0.066 | 0.94 | 5.98 |
| Constant | 0.27 | 0.13 | -2.75 | 0.006 | 0.11 | 0.69 |

**Backwards elimination of all variables**

No imputation on blinding status of statistician (N = 140)

| Significant finding reported | Odds ratio | Std. err. | z | P>\|z\| | [95% conf. interval] | |
| --- | --- | --- | --- | --- | --- | --- |
| **Statistician not blinded** | **0.78** | **0.33** | **-0.58** | **0.565** | **0.34** | **1.81** |
| Percentage of missing data | 0.94 | 0.02 | -2.63 | 0.008 | 0.89 | 0.98 |
| Clinicians not blinded | 4.15 | 2.13 | 2.78 | 0.006 | 1.52 | 11.35 |
| Multiple comparisons | 3.80 | 2.22 | 2.28 | 0.022 | 1.21 | 11.95 |
| Constant | 0.25 | 0.14 | -2.50 | 0.012 | 0.08 | 0.74 |

Sensitivity analysis - assuming that where the blinding status was unclear or no-response from authors the statistician was not blinded (N = 165).

| Significant finding reported | Odds ratio | Std. err. | z | P>\|z\| | [95% conf. interval] | |
| --- | --- | --- | --- | --- | --- | --- |
| **Statistician not blinded** | **0.92** | **0.36** | **-0.20** | **0.839** | **0.43** | **1.99** |
| Multiple comparisons | 3.57 | 1.82 | 2.50 | 0.012 | 1.32 | 9.67 |
| Percentage of missing data | 0.95 | 0.02 | -2.36 | 0.018 | 0.92 | 0.99 |
| Placebo | 0.26 | 0.13 | -2.68 | 0.007 | 0.10 | 0.70 |
| Constant | 0.78 | 0.32 | -0.59 | 0.555 | 0.35 | 1.76 |

**Forwards selection of all variables**

No imputation on blinding status of statistician (N = 140)

| Significant finding reported | Odds ratio | Std. err. | z | P>\|z\| | [95% conf. interval] | |
| --- | --- | --- | --- | --- | --- | --- |
| **Statistician not blinded** | **0.78** | **0.33** | **-0.58** | **0.565** | **0.34** | **1.81** |
| Clinicians not blinded | 4.15 | 2.13 | 2.78 | 0.006 | 1.52 | 11.35 |
| Percentage of missing data | 0.94 | 0.02 | -2.63 | 0.008 | 0.89 | 0.98 |
| Multiple comparisons | 3.80 | 2.22 | 2.28 | 0.022 | 1.21 | 11.95 |
| Constant | 0.25 | 0.14 | -2.50 | 0.012 | 0.08 | 0.74 |

Sensitivity analysis - assuming that where the blinding status was unclear or no-response from authors the statistician was not blinded (N = 165).

| Significant finding reported | Odds ratio | Std. err. | z | P>\|z\| | [95% conf. interval] | |
| --- | --- | --- | --- | --- | --- | --- |
| **Statistician not blinded** | **0.95** | **0.38** | **-0.13** | **0.899** | **0.44** | **2.06** |
| Journal | 0.28 | 0.18 | -1.94 | 0.052 | 0.08 | 1.01 |
| Multiple comparisons | 3.11 | 1.57 | 2.24 | 0.025 | 1.15 | 8.37 |
| Percentage of missing data | 0.96 | 0.02 | -1.97 | 0.049 | 0.93 | 1.00 |
| Outcome assessor not blinded | 2.06 | 0.78 | 1.92 | 0.055 | 0.98 | 4.32 |
| Constant | 1.70 | 1.45 | 0.62 | 0.535 | 0.32 | 9.02 |
