# Supplementary material for: Developing guidance for a risk-proportionate approach to blinding statisticians within clinical trials: a mixed methods study
Source: Trials. 2023 Jan 31;24:71. doi: 10.1186/s13063-022-06992-5 (PMC9887916; doi:10.1186/s13063-022-06992-5)
Supplement: Supplementary file 5 — Additional file 5. BOTS stakeholder survey responses. [file 13063_2022_6992_MOESM5_ESM.docx]

**Additional file 5 – BOTS Stakeholder Survey Responses**

Data download 02-Nov-2021 9.15am (N= 16 responses)

**Summary of provisional statements by category**

| **Category** | **Rule** | **N** |
| --- | --- | --- |
| High agreement | >70% agree (score 7,8,9) | 10 |
| Low agreement | >50% agree (score 7,8,9) | 7 |
| No agreement | No majority for agree or disagree | 21 |
| Low disagreement | >50% disagree (score 1,2,3) | 2 |
| High disagreement | >70% disagree (score 1,2,3) | 0 |

| **Question** | **Responses** | **Mean score** | **Percentage agreement** | **Percentage disagreement** | **Consensus** |
| --- | --- | --- | --- | --- | --- |
| 1.1) The trial statistician should not produce the randomisation list/codes. | 15 | 4.9 | 26.7 | 33.3 | No agreement |
| 1.2) The statistician(s) drafting or reviewing the statistical analysis plan should remain blinded prior to the statistical analysis plan being approved. | 16 | 7.2 | 75.0 | 0.0 | Consensus agree |
| 1.3) The trial statistician can request access to treatment allocation codes after the statistical analysis plan being approved, prior to database lock. | 16 | 4.4 | 25.0 | 37.5 | No agreement |
| 1.4) The trial statistician should not have access to treatment allocation codes until after the database has been locked. | 16 | 5.9 | 62.5 | 25.0 | Near consensus agree |
| 1.5) The trial statistician should remain blinded until after they have completed programming the final analysis | 16 | 5.5 | 50.0 | 31.3 | No agreement |
| 1.6) If possible, maintain the blind of all statisticians until the end of the study (for instance, if there are no interim analyses requiring disaggregate data). | 16 | 5.3 | 50.0 | 31.3 | No agreement |
| 2.1) It is important that the trial statistician attends the closed session of the Data Monitoring Committee (DMC). | 16 | 5.6 | 50.0 | 31.3 | No agreement |
| 2.2) It is beneficial to have at least two statisticians to prepare a Data Monitoring Committee (DMC) report (1) One non-blinded who conducts analysis by treatment group (2) One blinded involved in day-to-day management. | 16 | 6.1 | 56.3 | 12.5 | Near consensus agree |
| 2.3) A statistician should remain blinded if they could impact or influence data collection or recruitment (e.g., via participation in or contribution to the discussions within the Trial Management Group (TMG)). | 15 | 7.0 | 73.3 | 6.7 | Consensus agree |
| 2.4) The Data Monitoring Committee (DMC) requires disaggregate data (by treatment group) to fulfil its role adequately. | 16 | 6.8 | 62.5 | 12.5 | Near consensus agree |
| 2.5) Coded groups are more of a hindrance than a benefit for allowing the Data Monitoring Committee (DMC) to make effective decisions, (e.g., it can encourage the practice of guessing treatment groups, which risks inefficient or ineffective oversight). | 14 | 5.9 | 50.0 | 28.6 | No agreement |
| 2.6) If the focus of interim reports to the Data Monitoring Committee (DMC) are to describe data completeness or provide descriptive analyses (as opposed to comparative analyses) it is less important for the trial statistician to be blinded | 15 | 5.4 | 40.0 | 33.3 | No agreement |
| 2.7) For certain situations, blinded statisticians pose more of a risk to the trial (e.g., through suboptimal oversight and data monitoring) than having a non-blinded trial statistician. | 16 | 5.8 | 43.8 | 12.5 | No agreement |
| 2.8) For the trial statistician to conduct effective data cleaning and monitoring tasks, they need to be unblinded. | 16 | 4.8 | 25.0 | 31.3 | No agreement |
| 3.1) It is not necessary to blind statisticians for feasibility studies. | 16 | 5.5 | 43.8 | 25.0 | No agreement |
| 3.2) If there is a planned interim analysis, then it is preferable to have a blind and non-blinded statistician. | 16 | 5.9 | 43.8 | 18.8 | No agreement |
| 3.3) If a trial is open-label, it is not necessary to blind the trial statistician. | 16 | 5.3 | 37.5 | 25.0 | No agreement |
| 3.4) Pseudo-blinding the trial statistician (via the use of coded group names) is not feasible when there is an unequal allocation ratio. | 16 | 8.2 | 93.8 | 0.0 | Consensus agree |
| 3.5) For an adaptive trial where treatment groups may be added or dropped throughout the study (e.g. multi-arm multi-stage design) the trial statistician should not be blinded. | 16 | 5.1 | 31.3 | 25.0 | No agreement |
| 3.6) If the purpose of a trial is to apply for regulatory approval, it is important for the trial statistician to be blinded to treatment allocation. | 16 | 6.6 | 62.5 | 12.5 | Near consensus agree |
| 4.1) It is less important to blind statistician where the primary analysis is intention-to-treat (ITT) as opposed to, for example, a per protocol analysis – where a subset of randomised participants is analysed. | 15 | 4.5 | 13.3 | 46.7 | No agreement |
| 4.2) Trial statisticians should be unblinded for the purposes of monitoring treatment adherence. | 16 | 5.4 | 37.5 | 31.3 | No agreement |
| 4.3) It is impractical to blind trial statisticians if they are required to analyse mechanistic sub-studies or investigate the action of the treatment prior to the final analysis. | 16 | 6.3 | 50.0 | 12.5 | No agreement |
| 5.1) For interventions with a distinctive side effect profile, it is impractical to blind the statistician. | 16 | 6.8 | 87.5 | 12.5 | Consensus agree |
| 5.2) For low-risk interventions (where disaggregate data are not require for monitoring safety) unblinding the statistician before the end of the trial is not necessary. | 16 | 4.5 | 31.3 | 31.3 | No agreement |
| 6.1) The statistician is unlikely to be able to influence the reporting of simple-to-derive ‘hard’/objective outcomes. | 16 | 6.9 | 62.5 | 6.3 | Near consensus agree |
| 6.2) If the outcome involves combining data from multiple sources, there is potentially a greater risk associated with having a non-blinded trial statistician | 16 | 5.9 | 43.8 | 12.5 | No agreement |
| 6.3) Where ongoing analysis of safety outcomes is important, the trial statistician should not be blinded (either they will not have access to important safety data or will become unblinded because of safety data). | 16 | 5.8 | 37.5 | 6.3 | No agreement |
| 7.1a) If you need to blind a trial statistician, then you should maintain the blind by having rigorous processes for a blinded statistician to access/request data or treatment allocation. | 16 | 8.1 | 100.0 | 0.0 | Consensus agree |
| 7.1b) If you need to blind a trial statistician, then you should maintain the blind by ensuring that access to certain types of data is controlled/limited to non-blinded statisticians or other trial team members. | 16 | 7.3 | 87.5 | 12.5 | Consensus agree |
| 7.1c) If you need to blind a trial statistician, then you should maintain the blind by giving the responsibility to data management (or another team) to prepare reports and strip out data which could unblind. | 16 | 5.7 | 50.0 | 25.0 | No agreement |
| 7.1d) If you need to blind a trial statistician, then you should maintain the blind by ensuring the randomisation list/system is generated and maintained by another statistician or a separate team. | 14 | 6.4 | 57.1 | 21.4 | Near consensus agree |
| 7.2) If you are unable to maintain the blind then it's a waste of resources. (e.g. more complex database builds, increased workloads for other teams, inefficiency created by incomplete datasets for trial statistician). | 15 | 6.5 | 60.0 | 6.7 | Near consensus agree |
| 7.3) Where a non-blinded statistician is involved, they should attend the Data Monitoring Committee (DMC) meetings and must have sufficient experience and knowledge of the trial and methods. | 16 | 7.3 | 81.3 | 0.0 | Consensus agree |
| 7.4) The trial statistician should remain blinded if data are required to be presented separately by treatment arm at any stage prior to the final analysis | 16 | 6.1 | 50.0 | 6.3 | No agreement |
| 7.5) The Data Monitoring Committee (DMC) and Trial Steering Committee (TSC) should be consulted as early as possible to ask if they intend to see disaggregate data at any point in the trial. | 16 | 7.5 | 81.3 | 0.0 | Consensus agree |
| 7.6) Where an unplanned disaggregate analysis is requested by the Data Monitoring Committee (DMC), the risk of unblinding the trial statistician should be assessed at the time the request is made. | 16 | 7.1 | 75.0 | 0.0 | Consensus agree |
| 8.1) The trial statistician should always be blinded to treatment allocation. | 16 | 4.1 | 18.8 | 56.3 | Near consensus disagree |
| 8.2) The trial statistician should never be blinded to treatment allocation. | 16 | 3.4 | 18.8 | 62.5 | Near consensus disagree |
| 8.3) The decision to blind or not blind the statistician should be based on the benefits and risks associated with a particular trial. | 16 | 8.1 | 93.8 | 0.0 | Consensus agree |
