# Supplementary material for: Developing guidance for a risk-proportionate approach to blinding statisticians within clinical trials: a mixed methods study
Source: Trials. 2023 Jan 31;24:71. doi: 10.1186/s13063-022-06992-5 (PMC9887916; doi:10.1186/s13063-022-06992-5)
Supplement: Supplementary file 6 — Additional file 6. BOTS stakeholder group membership. [file 13063_2022_6992_MOESM6_ESM.docx]

**Additional file 6: BOTS stakeholder group membership**

| **Name** | **Role/Background** | **Institution** |
| --- | --- | --- |
| Mike Bradburn | Statistician | Sheffield Clinical Trials Unit |
| Catey Bunce | RM CTU Manager (Statistics & Training) | Royal Marsden Clinical Trials Unit |
| Mike Clarke | CTU Director | NI Clinical Trials Unit |
| Andrew Cook | Consultant in Public Health Medicine and Fellow in Health Technology Assessment | National Institute for Health and Care Research (NIHR),  Southampton Clinical Trials Unit |
| Heather Cook | Trial Manager | Exeter Clinical Trials Unit |
| Andy Fisher | Lead Senior Good Clinical Practice Inspector | Medicines and Healthcare products Regulatory Agency |
| Stephanie Foster | Clinical Trial Data Management Specialist | ICR Clinical Trials & Statistics Unit |
| Carrol Gamble | Statistician, CTU Director | Liverpool Clinical Trials Centre |
| Ruth Goodall | Statistician | MRC Clinical Trials Unit at UCL |
| Catherine Hewitt | Statistician, CTU Deputy Director | York Trials Unit |
| Steven Julious | Statistician | University of Sheffield |
| Edmund Juszczak | Statistician, previously CTU director | Nottingham Clinical Trials Unit |
| Alan Montgomery | Statistician, CTU Director | Nottingham Clinical Trials Unit |
| Khadija Rantell | Senior Statistical Assessor | Medicines and Healthcare products Regulatory Agency |
| Alison Steel | Senior Trial Manager | Newcastle Clinical Trials Unit |
| Matthew Sydes | Professor of Clinical Trials and Methodology | MRC Clinical Trials Unit at UCL |

**Meeting facilitators**

| **Name** | **Institution** |
| --- | --- |
| Christopher Partlett | Nottingham Clinical Trials Unit |
| Kirsty Sprange | Nottingham Clinical Trials Unit |
| Mais Iflaifel | Nottingham Clinical Trials Unit |
