# Supplementary material for: Developing guidance for a risk-proportionate approach to blinding statisticians within clinical trials: a mixed methods study
Source: Trials. 2023 Jan 31;24:71. doi: 10.1186/s13063-022-06992-5 (PMC9887916; doi:10.1186/s13063-022-06992-5)
Supplement: Supplementary file 7 — Additional file 7. BOTS guidance. [file 13063_2022_6992_MOESM7_ESM.docx]

**
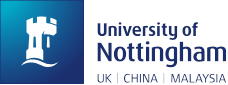

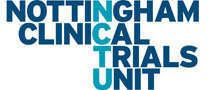
**

**Guidance for achieving a risk proportionate approach to blinding statisticians within clinical trials**

**Version 1.1**

**28-Sep-2022**

**
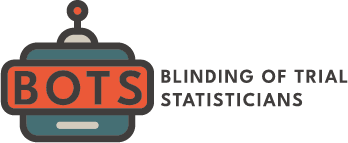

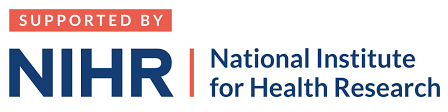
**

**Document History**

| Version number | Details/purpose of change | Authors/edited by | Date |
| --- | --- | --- | --- |
| 1.0 | New guidance for blinding statisticians in clinical trials. | See *Document details* on the next page. | 20-05-2022 |
| 1.1 | Minor revisions following peer review. Changed ‘statement’ to ‘considerations/ recommendations’ throughout. Amended section heading ‘Timing’ to say ‘Timing of unblinding’. | Mais Iflaifel  Christopher Partlett | 28-Sep-2022 |
|  |  |  |  |

**Document details**

| **Title:**  Guidance for achieving a risk proportionate approach to blinding statisticians within clinical trials |
| --- |
| **Authors:**  Mais Iflaifel^1^, Kirsty Sprange^1^, Jennifer Bell^2^, Andrew Cook^3^, Carrol Gamble^4^, Steven Julious^5^, Edmund Juszczak^1^, Louise Linsell^2^, Alan Montgomery^1^, Christopher Partlett^1^ |
| **Document purpose:**  The aim of this guidance is to provide for Clinical Trial Units (CTUs) points to consider (based on a risk proportionate approach) to blinding statisticians within clinical trials. |
| **Target audience:**  Researchers planning, designing, and conducting randomised trials |
| **Contact details:**  Christopher Partlett, PhD  Assistant Professor of Medical Statistics and Clinical Trials  chris.partlett@nottingham.ac.uk  Nottingham Clinical Trials Unit, University of Nottingham  Applied Health Research Building, University Park, Nottingham, NG7 2RD |

^1^ Nottingham Clinical Trials Unit, University of Nottingham, Nottingham, UK.

^2^ National Perinatal Epidemiology Unit, Nuffield Department of Population Health, University of Oxford, Oxford, UK

^3^ University of Southampton, Southampton, UK

^4^ Liverpool Clinical Trials Centre, University of Liverpool, Liverpool, UK

^5^ School of Health and Related Research, University of Sheffield, Sheffield, UK

**Abbreviations**

| **BOTS** | Blinding of Trial Statistician |
| --- | --- |
| **CI** | Chief Investigator |
| **CTU** | Clinical Trials Unit |
| **DMC** | Data Monitoring Committee |
| **NCTU** | Nottingham Clinical Trials Unit |
| **NS** | Non-blinded Statistician |
| **SS** | Second Statistician |
| **STS** | Senior Trial Statistician |
| **TMG** | Trial Management Group |
| **TS** | Trial Statistician |
| **TSC** | Trial Steering Committee |
| **UKCRC** | UK Clinical Research Collaboration |

Contents

[Introduction and rationale for the project 6](#_Toc94688348)

[Aims of the project 7](#_Toc94688349)

[Key definitions  7](#_Toc94688350)

[Considerations and recommendations 9](#_Toc94688351)

[Models for DMC interaction 15](#_Toc94688352)

[Benefits, risks and mitigation strategies to blind or not blind statisticians 16](#_Toc94688353)

# Introduction and rationale for the project

Historically, there has been limited empirical evidence to guide Clinical Trial Units (CTUs) and trial teams about the practice of blinding statisticians. A survey of the UK Clinical Research Collaboration (UKCRC) CTUs conducted in 2020 identified that there was considerable variation in practice when it came to blinding statisticians. Half the respondents mentioned that CTUs had a fixed approach to blinding statisticians rather than assessing the risk according to the trial circumstances.

CTUs can be broadly split into those that always blind the trial statistician (TS) and involve a second statistician for unblinded/disaggregate analyses, or those that maintain the blind until it is necessary to unblind the TS (*e.g.* for a Data Monitoring Committee (DMC) report). While there may be benefits to maintaining the blind of the TS, given the potential logistical and resource cost as well as other shortcomings of always blinding the statistician, it seems incongruent to apply this approach in all cases. This is particularly the case in open-label trials with subjective outcomes, where other risks of bias exist regardless of whether or not the trial statistician remains blinded.

While the current approaches are not based on empirical evidence, there are clearly benefits to developing an evidence-based risk proportionate approach for blinding statisticians in clinical trials. This inspired the Blinding of Trial Statisticians (BOTS) research team to develop guidance for CTUs on blinding the TS. BOTS employed a mixed methodology approach involving three parts:

I) a quantitative study to assess the impact of blinding statisticians on the proportion of trials reporting a statistically significant finding,

II) a qualitative study using focus groups to determine the perspectives of key stakeholders on blinding trial statisticians who work in the delivery and oversight of clinical trials,

III) Combining the results of parts I and II to develop a first draft of the provisional guidance statements. A stakeholder meeting with a group of expert stakeholders including statisticians, methodologists, trial and data managers, CTU directors, and unit managers, as well as members of independent trial oversight committees and representatives from the NIHR and the MHRA, was held to discuss the provisional guidance statements and produce the final guidance for CTUs.

# Aims of the project

The overall study aim is to provide recommendations on the practice of blinding statisticians. Objectives are:

- Compare reported outcomes between published studies with different blinding practices
- Understand current practice in academic CTUs, and reasons for these practices
- Understand stakeholder views on important risks and benefits to consider when deciding on blinding practice
- Produce guidance and a practical tool for CTUs to utilise a risk-based approach when considering blinding of statisticians

# Key definitions

| **Blinding (of a statistician)** | No access to allocated groups (including coded) or any data that might potentially unblind (*e.g.,* adherence or safety data). |
| --- | --- |
| **Interim analysis** | A formal between-group statistical analysis conducted prior to the final analysis. |
| **Interim DMC report** | Reports for the DMC containing trial data prior to the final analysis (this could contain, for example, (i) summary aggregate data only (ii) summary disaggregate data, or (iii) formal interim analyses. |
| **Non-blinded Statistician (NS)** | A statistician (separate to the trial management group) that is able to access data by allocation (and other data that may unblind). |
| **Open label study** | Clinicians and participants are aware of allocation. |
| **Pseudo-blinding (of a statistician)** | Access to coded treatment groups, but not labelled treatment groups. |
| **Second Statistician (SS)** | Responsible for validation of statistical analysis (*e.g.,* coding in parallel – independent of the TS). |
| **Senior Trial Statistician (STS)** | Responsible for oversight of the statistical methods. Does not generally handle or have access to the raw data for the trial. |
| **Trial Management Group (TMG)** | Responsible for day-to-day management of the trial. Multidisciplinary group that typically involves at least one statistician. |
| **Trial Statistician (TS)** | Responsible for the day-to-day statistical input into the trial. Conducts data cleaning, querying and analysis (usually under the supervision of an STS). |

# Purpose and scope

The aim of this guidance is to provide points to consider for researchers at CTUs in pursuit of achieving a risk proportionate approach to blinding statisticians within clinical trials. It is intended to be applied in trials where there is reasonable uncertainty on the most appropriate approach to blinding statisticians. While this applies to most trials conducted in the setting of academic CTUs, there might be a limited number of examples where the application of this guidance is not appropriate due to the constraints defined by regulatory bodies, funders or sponsors. For instance, if the purpose of a trial is to apply for market authorisation, it might not be appropriate for the trial statistician to be unblinded at any stage prior to the final analysis.

# Considerations and recommendations

**The decision to blind or not blind the statistician should be based on the benefits and risks associated with a particular trial.**

## Section 1: Timing of unblinding

| **Consideration/recommendation** | **Explanation** |
| --- | --- |
| 1.1) If the trial statistician is responsible for drafting or reviewing the statistical analysis plan, they should remain blinded prior to the statistical analysis plan being approved. | Finalising the analysis plan prior to unblinding mitigates against the risk of the trial statistician introducing risk via their selection of analysis sets or analysis methodology.  Subsequent revisions to the analysis plan should clearly document the changes, reasons for changes, and the timing of the changes in relation to the unblinding of the trial statistician. |
| 1.2) If the trial statistician is to be unblinded prior to the final analysis, then approving the statistical analysis plan prior to unblinding mitigates against some of the risk of the trial statistician introducing bias. |  |
| 1.3) Blinding the trial statistician up until the final database lock **and** approval of the statistical analysis plan effectively eliminates the risk that the trial statistician could introduce bias into the trial results. | Blinding prior to the final database lock theoretically prevents the trial statistician introducing bias by their: selection of analysis methodology, interactions with other members of the trial team, and conduct of other roles/responsibilities (e.g. querying and cleaning data).  One should also carefully consider the risks associated with prolonging the blind of the trial statistician (e.g., the resulting reduction in insight possessed by the trial statistician and the potential for less effective or ineffective oversight of the trial). |

## Section 2: Interaction with other groups

| **Consideration/recommendation** | **Explanation** |
| --- | --- |
| 2.1) The trial statistician should remain blinded if they could impact or influence data collection or recruitment. | It might be possible that the trial statistician is able to indirectly influence data collection or recruitment via interaction with researchers working on the trial.  Depending on its composition, this could happen via participation in or contribution to the discussions within the of Trial Management Group (TMG). |
| 2.2) It is important to consider how the blinding status of the trial statistician impacts on interactions between the trial team and the DMC. | Where resources permit, it is potentially beneficial to blind the trial statistician **and** involve a non-blinded statistician who conducts analysis by allocation (but who is otherwise independent of the trial).  However, it is vitally important that the non-blinded statistician has sufficient experience and knowledge of the trial and methods to attend meetings and support the DMC in providing adequate oversight for the trial.  Without suitable mitigation, maintaining the blind of the trial statistician may pose more of a risk to the integrity of the trial (e.g., through suboptimal oversight and data monitoring) than unblinding the trial statistician at an appropriate point (for instance, after approval of the statistical analysis plan).  As previously noted by DAMOCLES* the benefit of a statistician independent of the trial is that it maintains the principle of keeping blind all those involved with the trial. However, they also note that analysis often requires knowledge of the disease, the trial and detailed aspects of data collection. This potential loss of insight means that this approach is not recommended in general. |
| 2.3) Blinding the trial statistician and DMC members by presentation of coded groups may promote ineffective or inefficient oversight of the trial. | The presentation of coded treatment groups to the DMC is often not a robust method of blinding (for instance, depending on the safety profile of the intervention or the nature of other data provided). Coded groups can also promote ‘blinded’ members of the DMC to consciously or unconsciously guess the true allocation.  As noted by DAMCOLES* there are a number of issues with blinding the DMC in this way and, crucially, the practice is unlikely to improve participant safety. |

***** Grant AM, Altman DG, Babiker AB, Campbell MK, Clemens FJ, Darbyshire JH, et al. Issues in data monitoring and interim analysis of trials. Health Technol Assess. 2005;9(7):1-238.

## Section 3: Study design

| **Consideration/recommendation** | **Explanation** |
| --- | --- |
| 3.1) There is greater risk associated with not blinding the trial statistician where there is subjectivity in the selection of analysis sets. | For example: Where the primary analysis is intention-to-treat (ITT) there is little risk of the statistician influencing the inclusion or exclusion of participants from the analysis. For analysis that involve selecting a subset of randomised participants there is a greater risk of bias. |
| 3.2) It is not always appropriate or feasible to blind the trial statistician using coded groups. | For example: when there is an unequal allocation ratio or a distinctive side effect profile.  As noted above and by DAMOCLES coded groups are often not a robust or effective method of blinding. |
| 3.3) For open label studies, there is potentially less benefit to maintaining the blind of the trial statistician. | While the trial statistician is usually the only member of the trial team aware of accumulating data, when other members of the trial management team are unblinded, this weakens the argument for maintaining the blind of the trial statistician. |
| 3.4) For a feasibility trial, depending on the aims of the study, it may be less beneficial to blind the trial statistician. | Where the primary aim of a feasibility study is to demonstrate, for example, feasibility of recruitment, the risk of bias arising from an unblinded statistician is likely minimal. |
| 3.5) It may not be necessary or advantageous to maintain the blind of the trial statistician for an adaptive trial where interventions may be added or dropped throughout the study. | For example, in a platform trial or a multi-arm multi-stage design, it might be necessary to report the findings for several interventions sequentially, requiring the trial statistician to be unblinded. Blinding may also be impractical due to, for example, differential recruitment or duration of treatment. |

## Section 4: Types of intervention

| **Consideration/recommendation** | **Explanation** |
| --- | --- |
| 4.1) Depending on the type of interventions, there may be additional challenges and barriers to blinding the trial statistician. | For example, an intervention may have a distinctive side-effect profile, require intervention-specific data collection, or impact on other data (e.g. biomarkers). If access to these data is not restricted, then they may potentially unblind the trial statistician. |
| 4.2) For low-risk interventions, unblinding the statistician before the end of the trial may not be necessary. | For trials where a DMC is not required and disaggregate data are not required for monitoring safety, unblinding the trial statistician before the final analysis may not be necessary. |

## Section 5: Type of outcomes

| **Consideration/recommendation** | **Explanation** |
| --- | --- |
| 5.1) If the trial statistician is likely to become unblinded during the trial, there may be a greater risk of the trial statistician introducing bias for outcomes which are complex-to-derive or involve combining data from multiple sources. | This risk can be mitigated by detailed pre-specification of the derivations in the statistical analysis plan.  For trials involving data-linkage and combining data from multiple sources, it may be necessary to include additional details of how data will be combined (for example, how discrepancies are managed) in a separate document to the SAP, which should also be approved prior to unblinding the trial statistician.  Where the statistician is blinded, there may be an increased risk of error in the derivations – this risk can be mitigated by independent programming by a second statistician. |
| 5.2) Where ongoing analysis of safety outcomes by allocation is necessary, it might not be appropriate to blind the trial statistician. | If the trial statistician is to be unblinded it is important that the statistical analysis plan is approved prior to unblinding.  If the trial statistician is to be blinded, it is advisable to have another statistician with sufficient experience and knowledge of the trial to take on this role. |

## Section 6: Additional roles and responsibilities

| **Consideration/recommendation** | **Explanation** |
| --- | --- |
| 6.1) When considering the practicalities of blinding the trial statistician, consider whether any of the other roles/responsibilities of the trial statistician might potentially necessitate the statistician being unblinded. | These other roles and responsibilities might include analysis of sub-studies, analysis of safety data, or monitoring treatment adherence.  If the statistician is to be unblinded, it is important that the statistical analysis plan is approved prior to unblinding.  If the trial statistician is to be blinded, it is advisable to have another statistician or another team to take responsibility for those roles. |
| 6.2) If the trial statistician is to have primary responsibility for monitoring treatment adherence, it is likely to be beneficial or perhaps necessary for them to be unblinded. | In many cases, access to these data is likely to lead to de-facto unblinding.  Even where it is not essential to have knowledge of treatment allocation the statistician is likely to benefit from the additional insight afforded by awareness of treatment allocation. |
| 6.3) Effective data cleaning and monitoring may require knowledge of randomised allocation. If the trial statistician has primary responsibility for these tasks, it may be more beneficial for them to be unblinded. |  |
| 6.4) If the trial statistician is responsible for producing the randomisation list/codes, then it is recommended a second statistician implements the code and sets the random seed. | The generated list should be stored securely with restricted access to prevent the trial statistician becoming unblinded. It is vitally important that the custodian of the allocation lists is not directly or indirectly involved in recruitment of participants. |

## Section 7: Practicalities

| **Consideration/recommendation** | **Explanation** |
| --- | --- |
| 7.1) The resources required to maintain the blind of the trial statistician need to be proportionate to the perceived benefit to justify blinding the statistician. | Blinding the trial statistician requires resource and so the benefits need to outweigh the potential disadvantages. |
| 7.2) If the trial statistician is to be blinded, it is essential that rigorous processes are in place to maintain blinding. | For example, access to allocation and other potentially unblinding data is restricted to non-blinded statisticians or other trial team members. There should also be clear documentation, document history, and audit trail for a blinded statistician to access/request allocation data or unblinding datasets. |

# Models for DMC interaction

The table below summarises the different models for DMC interaction, the risk associated with each and suggestions to mitigate the risk.

| **Model** | **Risk** | **Mitigation** | **Comments** |
| --- | --- | --- | --- |
| Trial statistician (TS) unblinded. | Bias, or the perception of bias, caused by a member of the trial team being unblinded. | Approve SAP prior to unblinding.    Strictly limit the role of the TS in decision making (e.g., issues affecting the protocol or SAP) following unblinding. | Where applicable, clearly document in the SAP and/or protocol (who was unblinded and when) |
| TS and DMC both “blinded” using coded groups (pseudo-blinding). | The presentation of coded treatment groups to the DMC is often not a robust method of blinding.    Blinding of the DMC potentially hampers effective and accurate decision making. | Ensure there is an efficient and robust method for unblinding the DMC members where necessary.    (e.g. provide a sealed envelope with the treatment decodes). | As noted by DAMCOLES there are a number of issues with blinding the DMC in this way and, crucially, the practice is unlikely to improve participant safety. |
| TS blinded and descriptive disaggregate data/information provided by another team (e.g. data management or programmers). | Limited dialogue between the TS and the DMC, leading to potential for suboptimal or delayed decision-making owing to lack of insight. | Where possible, encourage dialogue within the open session, while maintaining the blind of the TS. | Only possible where descriptive data or information is provided to the DMC. Not possible if more advanced statistical analysis is required.  Caution required to avoid inadvertently unblinding the TS. |
| Programs provided to the independent statistician on the DMC by blinded TS. The DMC statistician then creates the report using allocation data (provided separately). | Limited dialogue between the TS and the DMC, leading to potential for suboptimal or delayed decision-making owing to lack of insight.    Independent statistician unlikely to have detailed knowledge of trial conduct/progress. | Where possible encourage dialogue within the open session, while maintaining the blind of the TS. | Caution required to avoid inadvertently unblinding the TS. |
| Blinded TS with report generated by a non-blinded statistician. | Limited dialogue between the TS and the DMC, leading to potential for suboptimal or delayed decision-making owing to lack of insight.    Non-blinded statistician may not possess the same detailed knowledge of trial conduct/progress. | Where possible encourage dialogue within the open session, while maintaining the blind of the TS.  Ensure that the non-blinded statistician has suitable experience and knowledge of the trial. | Caution required to avoid inadvertently unblinding the TS. |

# Benefits, risks and mitigation strategies to blind or not blind statisticians

Based on our analysis for the participants’ perceptions in the focus groups, Table 1 below summarises the benefits, risks, processes and suggested mitigation strategies for blinding and not blinding statisticians.

| Blinding | Not blinding |
| --- | --- |
| Benefits | |
| - TS can contribute freely to trial management/protocol discussions. - Enhancing credibility and quality of the trial by decreasing the possibility or perception of unconscious bias. - SAP can be authored, reviewed, and revised without a potential risk of introducing bias in the planned analysis. - TS can oversee or conduct day-to-day involvement in the trial without risk of introducing bias. - Reduces the potential for performance bias by maintaining confidentiality with people who do not know the emerging results (*e.g*., CI(s), PIs, TMG members, co-investigators, treating clinicians). - Reduces the possibility of pressure on statisticians to reveal findings (whether inadvertent or deliberate). | - Permits understanding of data in context and more insightful input to the trial (*e.g.,* clinical and safety decisions or stop/go decisions). - Facilitates more insightful conversations (with the DMC or with the TMG in an open-label study). - Decreases the risk of sub-optimal and delayed decision making and tenuous assumptions made about the data. - Allows the TS to effectively monitor sample size or analysis assumptions (*e.g*., standard deviations and event rates by group). - Greater insight into the data leading to higher quality analysis. - Permits more efficient and confident decision making. - Increases the ability to react more quickly and appropriately when safety issues arise. |
| Harm/Risk | |
| - Risk that maintaining the blind makes the trial processes unnecessarily inefficient, especially in open-label trials. - Some data may need to be concealed from the TS to prevent unblinding which may negatively impact data processing. - Inefficient or less effective oversight of the data if TS is not able to participate in closed session or in meaningful dialogue with the DMC. - Pseudo-blinding (using coded groups) can lead to less effective and inefficient oversight or monitoring. - Lack of understanding and insight into the trial context that might negatively impact the conduct and final analysis. | - TS may introduce bias by allowing knowledge of allocations to influence trial conduct (*e.g.,* through interaction with TMG/TSC). - TS may allow knowledge of allocation to influence the analysis (*e.g.*, through choice of analysis populations). |
| Mitigation | |
| - Involvement of an independent non-blinded statistician (with sufficient knowledge and experience) to interrogate potentially unblinding data, analyse data by allocation, and attend the closed session of the DMC. - Clearly document and communicate the blinding status of different roles within the trial to guard against unintentional unblinding of blinded team members. | - Approve first version of SAP prior to unblinding. Clearly document any changes (and reasons for change) following approval, who made them and their blinding status. - Limit TS’s interaction with other groups involved in decision making (*e.g*., TSC). - Keep unblinded and blinded members of the trial team separate. - Clearly document and communicate the blinding status of different roles within the trial to guard against unintentional unblinding of blinded team members. - Training for the unblinded TS not to reveal either knowledge of allocation or accumulating results. |
| Processes | |
| - The authoring and approval of the SAP can be conducted at a later stage. - IT processes must be in place to prevent unblinding (*e.g.,* may involve creation of blinded/unblinded datasets). - May require involvement of an additional non-blinded statistician who performs analyses by allocation and handles any potentially unblinding data. - Discuss with the DMC whether disaggregate results are necessary. - Blinded statistician attends only the open session of DMC meetings. - A separate non-blinded statistician attends the DMC closed session. - Monitoring of treatment adherence and safety can be conducted by other disciplines to maintain the TS blind. | - SAP must ideally be drafted, reviewed, and approved earlier in the trial (prior to unblinding). - Provide support for statistician if pressured to reveal allocation data/emerging results (*e.g.,* training, raising awareness, reporting systems, and setting ground rules for other TMG members). |

 TS – Trial Statistician
