# Supplementary material for: Developing guidance for a risk-proportionate approach to blinding statisticians within clinical trials: a mixed methods study
Source: Trials. 2023 Jan 31;24:71. doi: 10.1186/s13063-022-06992-5 (PMC9887916; doi:10.1186/s13063-022-06992-5)
Supplement: Supplementary file 8 — Additional file 8. BOTS Risk Assessment Tool (BRAT). [file 13063_2022_6992_MOESM8_ESM.docx]

**Additional file 8: BOTS Risk Assessment Tool (BRAT)**

| **Name of study or acronym** |  | |
| --- | --- | --- |
| **Research category**  *Tick all that apply* | Clinical Trial of an Investigational Medicinal Product (CTIMP) and/or medical device | |
|  | Clinical trial of a complex intervention | |
|  | Clinical trial of a surgical intervention | |
|  | Other | |
| **Trial’s key features** | Trial design |  |
|  | Primary outcome(s) |  |
|  | Blinding | *Describe the blinding of other individuals in the trial (where confirmed)* |
|  | Other comments | *e.g. Does the trial involve the collection of any potentially unblinding data?* |
| **Timing of form completion** | Funding application stage | |
|  | Study set-up | |
|  | Study delivery (any review conducted during the trial) | |

**Introduction**

This template is intended to be completed having considered the supporting guidance document: *Guidance for blinding statisticians in clinical trials version 1.0*.

**Purpose**

- The main aim of this tool is to help inform the discussions and decision regarding the blinding status of statistician(s) for a given trial.
- This tool could also be used as a record for Medicines and Healthcare products Regulatory Agency (MHRA) or Audit inspections to demonstrate and justify the decisions made.

**Procedure**

- Once the fundamental design features of a trial have been determined, a member of study team (preferably a statistician) should complete the Risk Assessment Tool (see Appendix A).
- The Risk Assessment Tool can be completed at different stages, e.g., prior to securing funding which may be more important for smaller units, or at designing/setting-up phase to help with allocation of staff for larger units with bigger teams.
- If the protocol undergoes substantial changes (or it becomes necessary to look at unblinded data) once the risk assessment form has been completed, this form may be required to be updated.

**How to complete the tool**

- The tool provides a framework to identify the risks associated with both blinding or not blinding the statistician
- The tool is split into 6 domains and each domain includes rows with suggestions for potential risks
- These rows are not intended to be exhaustive and there are blank rows included to add other possible risks

| **Factors to consider in the decision to blind the TS** | Potential risks associated with either blinding or not blinding the statistician |
| --- | --- |
| **Likelihood and risk to the trial** | The potential risk to the trial (either low, medium or high). This rating should encompass both the likelihood and the severity the risk. |
| **Comments** | A space to explain your assessment of the risk |
| **Mitigation** | Strategies that might be employed to mitigate the risk |

**Appendix A**

| **Risks associated with blinding the trial statistician** | | | | | | **Risks associated with not blinding the trial statistician** | | | | |
| --- | --- | --- | --- | --- | --- | --- | --- | --- | --- | --- |
| **Factors to consider in the decision to blind the TS?** | Likelihood | | Risk to the trial | Comments Explain your choice for the assessment of the risk | **Mitigation**  What might be done to mitigate the risk? | Factors to consider in the decision to not blind the TS? | **Likelihood** | **Risk to the trial** | Comments Explain your choice for the assessment of the risk | **Mitigation**  What might be done to mitigate the risk? |
|  | L = LowM = MediumH = High | | |  |  |  | L = LowM = Medium **H = High** | |  |  |
| **Section 1 – Interaction with other groups**  **Note: Please consider the following points while completing this section**   - **TS’s impact or influence on data collection or recruitment (e.g., via TMGs)** - **Interaction with the DMC** - **Risks of coded A/B groups. Guessing treatment groups – guessing incorrectly or ineffective oversight** | | | | | | | | | | |
| Could the blinded statistician adversely affect the trial oversight as they are unable to present closed report to the DMC? |  |  | |  |  | Could the trial statistician have an influence directly or indirectly on data collection or recruitment? |  |  |  |  |
| Could the use of coded group names (e.g., A/B) compromise the blinding of the statistician? |  |  | |  |  | Could the scientific integrity of the study be compromised by a non-blinded statistician interacting with other members of the study team? |  |  |  |  |
| Could the use of coded group names lead to ineffective oversight? |  |  | |  |  |  |  |  |  |  |
|  |  |  | |  |  |  |  |  |  |  |
| **Section 2 – Study design**  **Note: Please consider the following points while completing this section**   - **Open label vs blinded randomised clinical trials** - **Feasibility trials** - **Complex trials (e.g., multi-arm, platform, adaptive or multi-arm multi-stage or early phase RCTs, umbrella studies)** - **Interim analyses** - **Analysis sets (e.g. Intention to treat, per protocol)** | | | | | | | | | | |
| Might the trial statistician be required to analyse disaggregate before the end of the study? |  |  | |  |  | Could the non-blinded statistician unblind other members of the study team? |  |  |  |  |
| Could the blinded trial statistician be potentially unblinded by other members of the study team? |  |  | |  |  | Could the non-blinded trial statistician influence the planned analysis (after being unblinded)? |  |  |  |  |
| Could any analyses (e.g. planned or unplanned interim analyses) be compromised by lack of knowledge of treatment allocation? |  |  | |  |  | Could the non-blinded trial statistician influence the analysis sets? (after being unblinded) |  |  |  |  |
|  |  |  | |  |  |  |  |  |  |  |
| **Section 3 – Interventions**  **Note: Please consider the following points while completing this section**   - **Low-risk or high-risk intervention (consider the value of presenting and reporting disaggregate data to the DMC)** - **Types of intervention (medical, surgical, complex)** | | | | | | | | | | |
| Could safety of participants be compromised by not presenting disaggregate data to the DMC? |  |  | |  |  |  |  |  |  |  |
| Could integrity and accuracy of the trial findings be compromised by not presenting disaggregate data to the DMC? |  |  | |  |  |  |  |  |  |  |
|  |  |  | |  |  |  |  |  |  |  |
| **Section 4 –Types of key outcomes**  **(e.g., adherence, safety, allocation-specific outcomes)** | | | | | | | | | | |
| Could the blinded trial statistician be unintentionally unblinded by any trial data? |  |  | |  |  | Could the non-blinded trial statistician influence the derivation of the outcome? |  |  |  |  |
| Could data integrity be compromised if the blinded trial statistician is not permitted to access certain trial data? |  |  | |  |  |  |  |  |  |  |
|  |  |  | |  |  |  |  |  |  |  |
| **Section 5 – Additional roles and responsibilities**  **(e.g. analysis of sub-studies, analysis of safety data, data cleaning or monitoring)** | | | | | | | | | | |
| Is there a chance that a blinded statistician is unable to effectively clean, monitor and query data? |  |  | |  |  | Could access to treatment allocation contribute to performance bias (e.g. differential monitoring and querying between treatment groups?) |  |  |  |  |
| Are there other roles and responsibilities of the trial statistician (e.g. monitoring treatment adherence) that would be adversely impacted by blinding? |  |  | |  |  |  |  |  |  |  |
|  |  |  | |  |  |  |  |  |  |  |
| **Section 6 – Practicalities**  **(e.g. availability of staff, experience of staff, funding, space in the unit)** | | | | | | | | | | |
| Are IT and data management processes adequate to maintain blinding of the trial statistician? |  |  | |  |  | Are IT and data management processes adequate to permit unblinding of the trial statistician while maintaining the blind of other members of the team? |  |  |  |  |
| Could the lack of (suitably experienced) statisticians compromise the quality of oversight if additional statisticians are required for interim reports? |  |  | |  |  | Could logistical issues prevent unblinding the statistician (e.g., if working in close proximity with blinded team members)? |  |  |  |  |
| Could logistical issues prevent effective blinding of the statistician (e.g., if working in close proximity with non-blinded team members)? |  |  | |  |  |  |  |  |  |  |
| Is there insufficient funding to cover the time of a blinded and non-blinded statistician? |  |  | |  |  |  |  |  |  |  |

| **The overall risk associated with blinding the TS is** | | | **The overall risk associated with not blinding the TS is** | | |
| --- | --- | --- | --- | --- | --- |
| Low | Medium | High | Low | Medium | High |

| **Decision**  Include the final decision regarding the approach to blinding the trial statistician, with justification and detail any mitigation strategies to be employed. |
| --- |

**Form completed by**

| **Name** |  | **Role** |  |
| --- | --- | --- | --- |
| **Signature** |  | **Date** | DD-MMM-YYYY |
